# Pest status, molecular evolution, and epigenetic factors derived from the genome assembly of *Frankliniella fusca*, a thysanopteran phytovirus vector

**DOI:** 10.1186/s12864-023-09375-5

**Published:** 2023-06-22

**Authors:** Michael A. Catto, Paul E. Labadie, Alana L. Jacobson, George G. Kennedy, Rajagopalbabu Srinivasan, Brendan G. Hunt

**Affiliations:** 1 Department of Entomology, University of Georgia, Athens, GA 30602 USA; 2 Department of Entomology and Plant Pathology, North Carolina State University, Raleigh, NC 27695 USA; 3 Department of Entomology and Plant Pathology, Auburn University College of Agriculture, Auburn, AL 36849 USA; 4 Department of Entomology, University of Georgia, Griffin, GA 30223 USA

**Keywords:** Crop pest, Thrips, Orthotospovirus, Selection pressures, CpGo/e, i5K

## Abstract

**Background:**

The tobacco thrips (*Frankliniella fusca* Hinds; family Thripidae; order Thysanoptera) is an important pest that can transmit viruses such as the tomato spotted wilt orthotospovirus to numerous economically important agricultural row crops and vegetables. The structural and functional genomics within the order Thysanoptera has only begun to be explored. Within the > 7000 known thysanopteran species, the melon thrips (*Thrips palmi* Karny) and the western flower thrips (*Frankliniella occidentalis* Pergrande) are the only two thysanopteran species with assembled genomes.

**Results:**

A genome of *F. fusca* was assembled by long-read sequencing of DNA from an inbred line. The final assembly size was 370 Mb with a single copy ortholog completeness of ~ 99% with respect to Insecta. The annotated genome of *F. fusca* was compared with the genome of its congener, *F. occidentalis*. Results revealed many instances of lineage-specific differences in gene content. Analyses of sequence divergence between the two *Frankliniella* species’ genomes revealed substitution patterns consistent with positive selection in ~ 5% of the protein-coding genes with 1:1 orthologs. Further, gene content related to its pest status, such as xenobiotic detoxification and response to an ambisense-tripartite RNA virus (orthotospovirus) infection was compared with *F. occidentalis*. Several *F. fusca* genes related to virus infection possessed signatures of positive selection. Estimation of CpG depletion, a mutational consequence of DNA methylation, revealed that *F. fusca* genes that were downregulated and alternatively spliced in response to virus infection were preferentially targeted by DNA methylation. As in many other insects, DNA methylation was enriched in exons in *Frankliniella*, but gene copies with homology to DNA methyltransferase 3 were numerous and fragmented. This phenomenon seems to be relatively unique to thrips among other insect groups.

**Conclusions:**

The *F. fusca* genome assembly provides an important resource for comparative genomic analyses of thysanopterans. This genomic foundation allows for insights into molecular evolution, gene regulation, and loci important to agricultural pest status.

**Supplementary Information:**

The online version contains supplementary material available at 10.1186/s12864-023-09375-5.

## Background

**Fig. 1:**
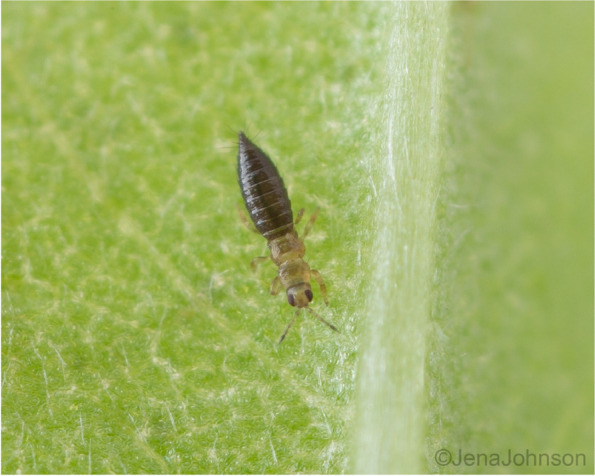
Tobacco thrips (*Frankliniella fusca* Hinds). Photo credit: Jena Johnson, Entomology Department, University of Georgia

The family Thripidae, which includes the genus *Frankliniella*, contains > 7,000 thrips species [1]. However, the tobacco thrips (*Frankliniella fusca* Hinds) (Fig. 1) genome presented in this paper represents only the third thysanopteran genome assembly published to date, after the melon thrips (*Thrips palmi* Karny) [2] and the western flower thrips (*Frankliniella occidentalis* Pergrande) [3].

Ecologically, *F. fusca* is native to North America and is a major agricultural pest [4]. However, it can also be found inhabiting three other continents [5–7], and its dispersal tends to vary by season [8, 9]. *F. fusca* is highly polyphagous and has been, for example, observed to feed on economically important plants in at least fifteen plant families including Amaryllidaceae, Fabaceae, Malvaceae, and Solanaceae [7]. The primary damage from the thrips feeding is limited to the plant epidermal cells, causing dark brown spots on the leaves, and inhibiting plant growth [5, 10]. *F. fusca* management on various crops has been a growing concern, as their populations have been developing resistance to insecticides [11].

While feeding damage can itself be detrimental to plants, the transmission of debilitating viruses to susceptible crops is of much greater concern [12–14]. Thrips, such as *F. fusca*, can transmit viruses belonging to the genus *Orthotospovirus* and family *Tospoviridae*. Approximately twenty species of orthotospoviruses are known and are exclusively transmitted by about a dozen thrips species in a persistent and propagative manner (the virus persists for life after acquisition in thrips and replicates within their tissues) [14]. Upon acquisition, the orthotospovirus traverses the midgut membrane barrier via receptor-mediated endocytosis and further translocates to other tissues including the salivary glands, where it replicates [15]. From the salivary glands, the virus is typically inoculated to plant hosts in the saliva during feeding [16–21]. The virus is also transmitted in a stage-dependent manner: for adult thrips to inoculate the virus successfully, they must acquire the virus as first or second instar larvae [20, 22]. This phenomenon is attributed to anatomical differences in tissue types between the stages [23–25]. Interestingly, tomato spotted wilt orthotospovirus (TSWV) acquisition by *F. fusca* has been shown to exhibit context-specific effects on fitness [14, 26–28]. The interactions between thrips and orthotospoviruses are intricate and indicate co-evolution [19, 29].

*F. fusca* along with *F. occidentalis* are the two most important orthotospovirus vector species in temperate and sub-tropical regions worldwide, whereas *T. palmi* is generally regarded as a tropical pest [7, 30, 31]. *F. fusca* and *F. occidentalis*, despite their congeneric status, exhibit ecological differences reflected by different abundances among host plant taxa [12]. In this study, the *F. fusca* genome was assembled and compared with the existing *F. occidentalis* genome to gain insights into the molecular evolution, expression [32], and epigenetics of factors associated with thrips-plant-virus trophic interactions.

## Results

### Genome assembly and annotations

**Fig. 2:**
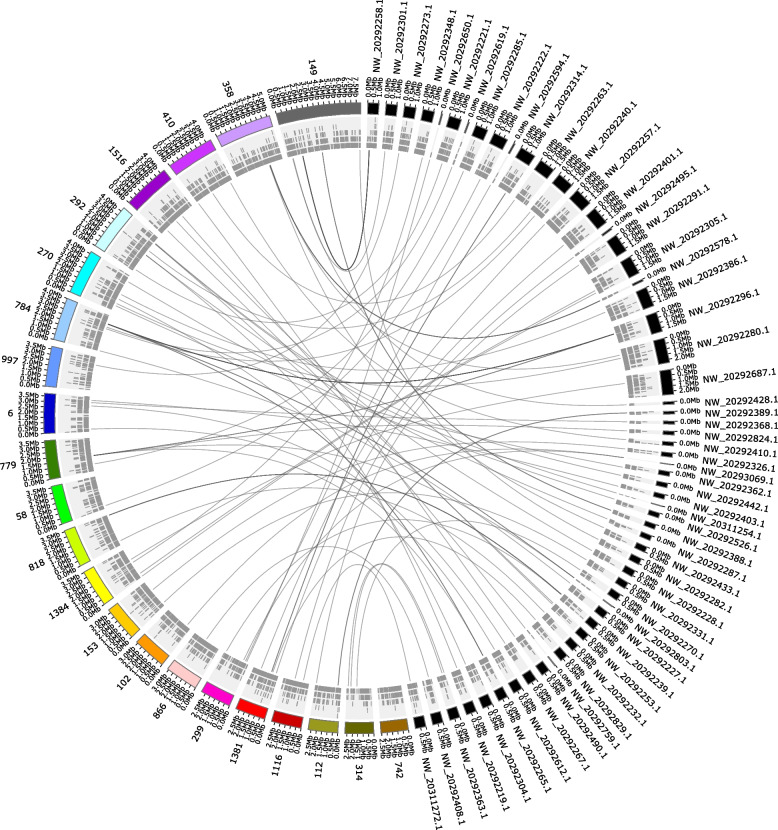
Visual comparison of two *Frankliniella* genomes. The *F. fusca* genome assembly (numeric only accessions) in comparison with the *F. occidentalis* genome assembly (NW accessions) [3]. Contigs of the *F. fusca* genome assembly ≥ 2.5 Mb in length were selected for visualization. Gene locations are shown below the respective assembly fragment. Regions that were ≥ 1 kb in length and ≥ 90 percent identity were linked

The *F. fusca* genome assembly contained 46 ungapped scaffolds measuring ~ 370 Mb (Table 1). The assembly length and N50 of the long-read based *F. fusca* genome indicated a more complete (1.4x) assembly compared with the earlier short-read based assembly of its congener *F. occidentalis* [3, 33] (Table 1; Fig. 2). Benchmarking Universal Single-Copy Orthologs (BUSCO) analysis was conducted to assess assembly completeness relating to inclusion of core Insecta genes [34]. The *F. fusca* genome assembly showed 99.0% completeness with 1,325 universal single copy and 29 duplicated orthologs. Universal single copy orthologs are defined as those genes present in over 90% of species within the class Insecta [34]. The *F. fusca* assembly was annotated with gene models using the MAKER pipeline (Additional file 1: Fig. S1 and Table S1) [35]. *F. fusca* gene annotations included 96.6% of the complete BUSCO Insecta sequences (Additional file 1: Fig. S2).

**Table 1:** Genome assembly statistics

	*Frankliniella fusca*	*Frankliniella occidentalis* [3]
Sequencing technology	PacBio + Illumina	Illumina
Genome coverage	113x	158.7x
Number of contigs	1,444	6,263
Total length of genome assembly (bp)	372,449,807	415,771,118
Total ungapped length of genome assembly (bp)	372,449,807	263,737,329
GC (%)	49.51	50.87
Scaffold N50 (bp)	1,182,854	948,890

### Phylogenetic context

**Fig. 3:**
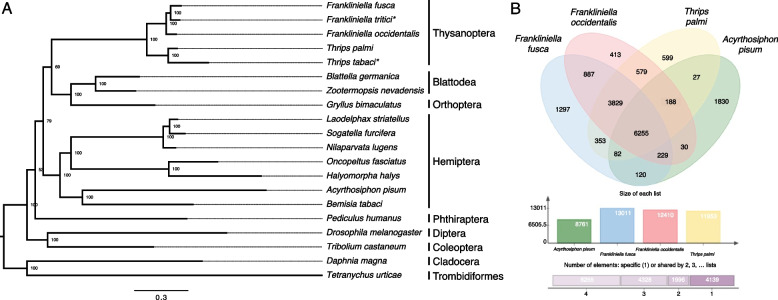
*F. fusca* phylogenetic relationships and orthology. **A** Species tree produced from ortholog comparisons across six insect orders and two additional non-insect arthropod orders (orders denoted at right; *de novo transcriptome data used for *F. tritici* and *T. tabaci*; scale bar denotes amino acid substitutions per site) [2, 3, 33, 42–59]. Clade bootstrap support values are labelled on the respective node. **B** Gene family orthologous clusters shared between four insect species. Venn diagram constructed from *F. fusca*, *F. occidentalis* [3]*, T. palmi* [2], and *A. pisum* [42]

Functional annotation of *F. fusca* gene models based on sequence homology was performed with the eggNOG database, which is comprised of the Kyoto Encyclopedia of Genes and Genomes (KEGG) pathways, Pfam domains, and gene ontology (GO) [36–41]. A total of 17,389 *F. fusca* proteins were assigned functional information (Additional file 2: Table S2). To infer *F. fusca* species relatedness, a consensus tree was generated from twenty arthropod species with high-quality genome assemblies including the congener *F. occidentalis*, three additional thysanopteran species (two based on de novo transcriptome assemblies), six non-thysanopterans in class Insecta, and two non-insect arthropods (Fig. 3A). An ortholog comparison between the *F. fusca* and *F. occidentalis* species assigned 87.3% of 26,761 *F. fusca* MAKER genes and 85% of 23,356 *F. occidentalis* RefSeq genes to 12,596 and 12,197 orthogroups respectively (Additional file 1: Table S3). There were 7,912 (1:1) orthologs identified between the two *Frankliniella* species using Orthofinder (Additional file 3: Tables S4 & S5).

Gene family membership was assigned to a set of four species (three thrips and the pea aphid) using OrthoVenn2 [60], which runs OrthoMCL [61]. A total of 6,255 gene families (orthologous clusters) were found to be shared between *F. fusca*, *F. occidentalis* [3]*, T. palmi* [2], and the pea aphid (*Acyrthosiphon pisum* Harris) [42] (Fig. 3B). Another 887 gene families were shared between the two *Frankliniella* species, of which 1,297 gene families were specific to *F. fusca* (Fig. 3B).

### Gene content relevant to pest status

The feeding behavior of thrips exposes them to a variety of xenobiotics including plant defensive chemicals (secondary metabolites) and insecticides [62, 63]. Thrips have developed mechanisms to efficiently metabolize xenobiotics [64]. Some of the relevant detoxification molecules include: Acyl-CoA desaturases [65], ATP-binding cassette (ABC) transporters [66], cathepsins [67], carboxylesterases (CE) [68], cytochrome P450 monooxygenases (CYP) [69], Dicer dimerization domains [70], glutathione S-transferases (GST) [71], heat shock proteins 70 & 90 (Hsp70 & Hsp90) [72], the mitogen-activated protein (MAP) kinases, and Jun N-terminal kinases (JNK) [73] (Additional file 4: Tables S6 & S7).

CYPs, CEs, and GSTs are known to detoxify plant xenobiotics such as phytochemicals and insecticides [69, 74, 75]. Forty-seven 1:1 orthologs of CYPs (IPR001128) were found between *F. fusca* and *F. occidentalis*. Fewer were detected among more distantly related species; 15, 15, and nine 1:1 orthologs of CYPs (IPR001128) were found between *F. fusca* and *A. pisum*, *F. fusca* and *Z. termopsis*, and *F. fusca* and *D. melanogaster,* respectively. CYPs can be taxonomically broken down into two classes (B-class and E-class), with E-class being found primarily within eukaryotes and itself subdivided into five groups [76]. Cytochrome P450, E-class, group I (IPR002401), is the largest of the five and contains CYPs specialized in the metabolism of exogenous substrates and physiologically active compounds. There were seventy-eight and ninety-one genes containing CYP E-class group I families in *F. fusca* and *F. occidentalis* assemblies, respectively. Out of the detoxification molecules, both thrips species were found to contain two CE (IPR003140) containing genes, which also were 1:1 orthologs. Additionally, eleven GST (IPR004045) 1:1 orthologs were identified.

Previously reported genes involved with thrips-plant-virus interactions include those involved with cell surface reception, virus replication, and innate immunity. Such genes include aminopeptidase N, endopeptidases, peroxiredoxins, peptidoglycan recognition proteins (PGRPs), lysozymes, trypsin, 40S ribosomal protein, and serine proteases [21, 32]. Notably, PGRPs detect and fight infection and initiate a humoral response of the Toll or Imd pathways [77]. Importin (IPR002652), a nuclear transport complex, may be involved in the binding of the virus to the cell membrane and control entry of the virus into the vector’s salivary glands [78], whereas cathepsins and peptidases may play a role in uptake of the virus into the vector [67, 79]. Glucose metabolism associated genes such as alpha glucosidase may inhibit binding and reduce replication of the virus within the vector [80]. Additionally, Hsp70 and Hsp90 were involved in inhibiting virus infection in vectors [72]. Heparan sulfate (IPR037359) genes are known to facilitate binding of the virus to the salivary cells in vectors [78].

**Fig. 4:**
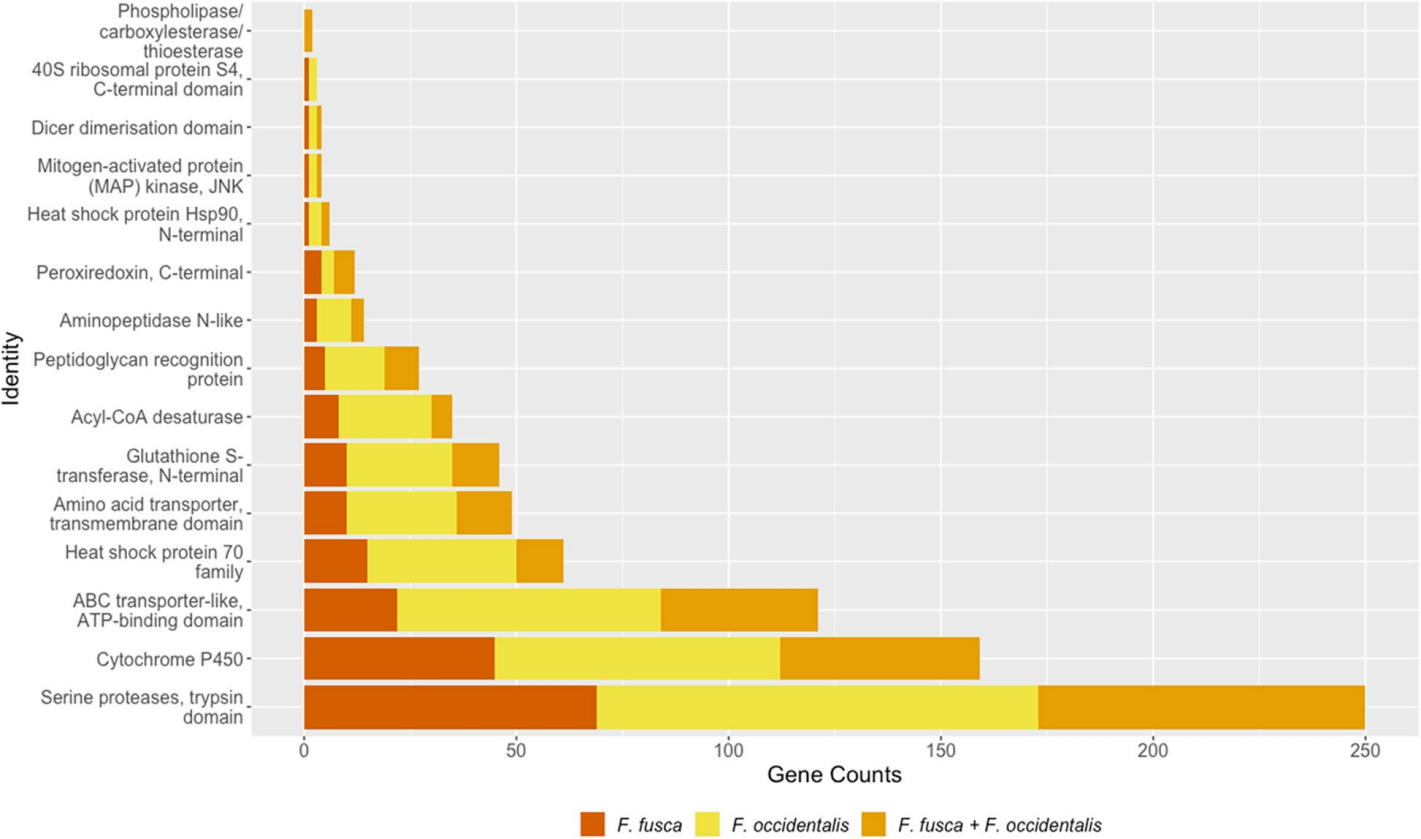
Detoxification and virus interaction related gene counts. Unique genes and non-1:1 orthologs are combined from *F. fusca* and *F. occidentalis* and compared with the 1:1 orthologs (*F. fusca* + *F. occidentalis*). Gene counts refers to the number of genes which contain a given protein domain identity. Genes may contain more than one of the given protein domains

The copy numbers of genes with evidence for molecular interaction between the two thrips vectors (*F*. *fusca* and* F*. *occidentalis*) and the virus revealed widespread variation between species (Fig. 4). On an average, higher copy number variation (CNV) of genes in relation to the pest status were documented in *F. occidentalis* (x̄ ≈ 40) than in *F*. *fusca* (x̄ ≈ 28); however, the mean counts were not statistically different (t-test, *p* = 0.2452).

### Molecular evolution of pest status genes

The availability of genome assemblies from *F. occidentalis* and *F. fusca* made it possible to assess rates of protein evolution following their divergence from a common ancestor and the ratio between the rate of non-synonymous to synonymous substitutions (dN/dS; Additional file 3: Tables S4 & S5). Variation in dN/dS can reflect differences in the strengths of positive selection or purifying selection. Genes with dN/dS > 1 when averaged across all codons were identified. A dN/dS > 1 ratio is generally considered a signal of positive selection operating on a locus [81]. Of the 7,817 assessed ortholog pairs detected from *F. occidentalis* and *F. fusca*, 388 (4.96%) exhibited values of dN/dS greater than one (Additional file 5: Tables S8-S11).

Assessment of molecular evolution in thrips further helped to address whether the selective constraints experienced by genes that respond to virus infection and detoxification differ from those that do not. Of the 223 genes with 1:1 orthologs belonging to fifteen InterProScan categories relating to pest status, five were found to have a dN/dS > 1: atrial natriuretic peptide-converting enzyme isoform X1 (FUS_00002678-RA), hypothetical predicted protein (FUS_00019172-RA), trypsin 3A1-like isoform X2 (FUS_00004482-RA), cytochrome P450 4d2-like (FUS_00010490-RA), and probable cytochrome P450 12a5, mitochondrial (FUS_00026641-RA).

Previously generated RNA-seq gene expression data from three life stages of *F. fusca*, with and without orthospovirus-infection [32], were mapped to the *F. fusca* reference assembly. As previously reported [32], most of the differential gene expression between orthotospovirus-viruliferous and non-viruliferous individuals occurred in larval and adult stages, with fewer differentially expressed genes (DEGs) detected at the pupal stage (Additional file 6: Tables S12-S14). Upon assessing the distributions of dN/dS values for DEGs and non-DEGs across the three developmental stages, it was found that there were significant differences in dN/dS for DEGs and non-DEGs as classified from the larval and adult stages, but there was not a consistent directional signal of elevated or reduced rates of protein evolution for those DEGs (Additional file 1: Fig. S4A & S4B). The proportion of genes with dN/dS > 1 among DEGs (based on differential expression in any stage) was 4.6% and among non-DEGs was 5.3%, which did not represent a significant difference (Fisher’s Exact Test, *p* = 0.1882).

**Fig. 5:**
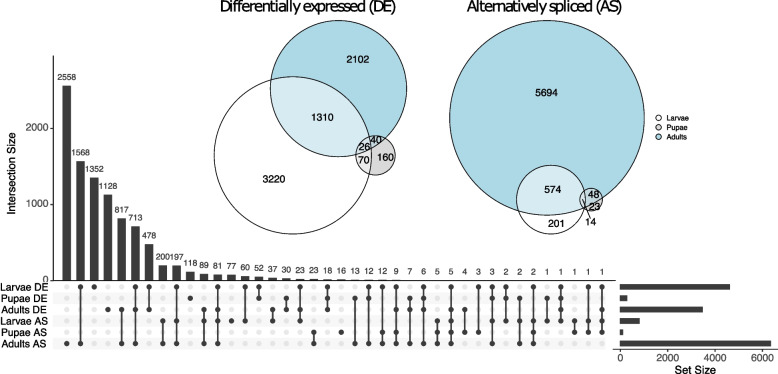
Overlap across *F. fusca* developmental stages for virus-responsive genes. Venn diagrams indicate overlap between developmental stages of differentially expressed (DE) genes (*n* = 6,928) and alternatively spliced (AS) genes (*n* = 6,554). Upset plot (below) indicating overlap of DE and AS genes

The presence of the *F. fusca* reference genome facilitated the analysis of alternative splicing of transcripts for the first time between orthotospovirus-viruliferous and non-viruliferous individuals from the previously generated RNA-seq data [32] using a metric of differential exon usage normalized by sequencing depth (Additional file 7: Tables S15-S17). Significant alternative splicing in response to orthotospovirus-infection, plant feeding, and detoxification was most prevalent in adults, and, as in the case of differential expression, alternative splicing was sparse in the pupal stage (Fig. 5). Upon assessing the distribution of dN/dS values for alternatively spliced (AS) and non-AS genes across the three developmental stages, it was found that there were significant differences in dN/dS for AS and non-AS genes as classified from the pupal and adult stages, but there was not a consistent directional signal of elevated or reduced rates of protein evolution for these comparisons (Additional file 1: Fig. S5A & S5B). The proportion of genes with dN/dS > 1 among AS genes (based on alternative splicing events in any stage) was 3.2% and among non-AS genes was 6.9%, indicating a significant depletion of genes with dN/dS > 1 among AS genes (Fisher’s Exact Test, *p* < 0.00001).

**Fig. 6:**
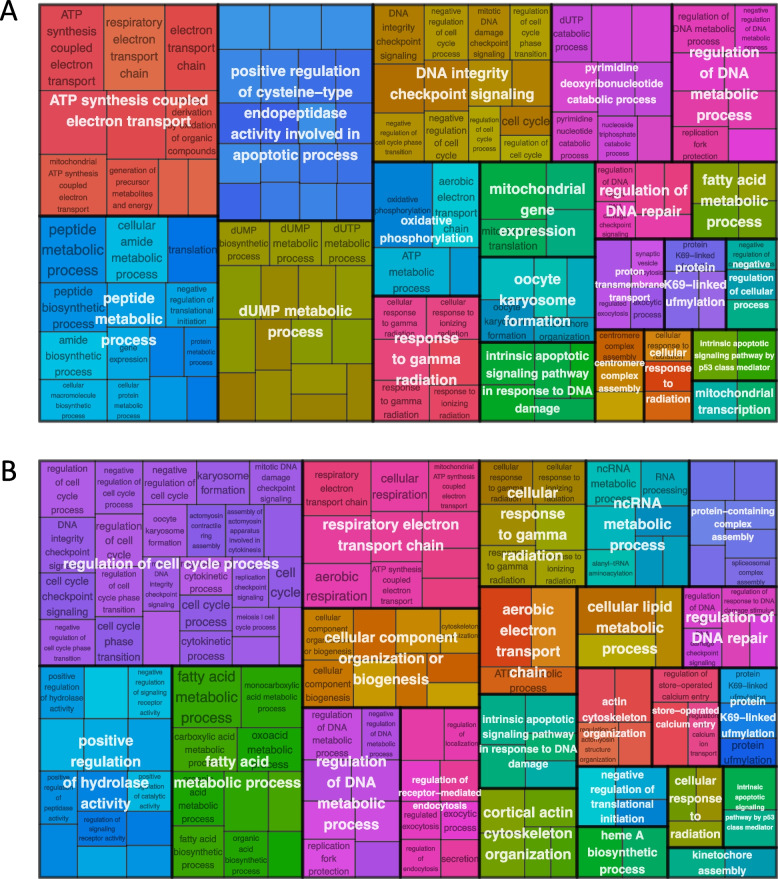
Tree map GO term enrichment for virus-responsive genes with dN/dS > 1. Biological processes category GO term distribution is shown for **A** differentially expressed genes and **B** alternatively spliced genes. Each individual box represents an enriched (*p* < 0.05) GO term, with one representative term per supercluster. The size of each box relates to the relative frequency of the respective GO term

Within the virus responsive genes, 170 DEGs and 134 AS genes within at least one developmental stage had dN/dS > 1, consistent with positive selection operating on these loci in the *Frankliniella* genus (Additional file 1: Fig. S3, Additional file 5: Tables S8 & S9). One of these genes was differentially expressed in all three developmental stages –putative metal responsive transcript (FUS_00001516-RA) and one was alternatively spliced in all three stages –cytochrome c oxidase subunit III (FUS_00005990-RA). The intersection between DEGs and AS genes revealed that 2,233 genes exhibited changes to both their expression levels and splicing patterns in response to orthotospovirus-infection in *F. fusca* when considering significant differences in any of the three developmental stages. Eighty-two genes with a dN/dS > 1 were both differentially expressed and alternatively spliced in response to orthotospovirus-infection (Additional file 5: Table S10). DEGs (Fig. 6A, Additional File 1: Fig. S6A), AS genes (Fig. 6B, Additional File 1: Fig. S6B), and the intersection between DEGs and AS genes (Additional File 1: Fig. S7 & Fig. S8) with dN/dS > 1 were checked for GO term enrichment within the biological process (BP) category of GO terms. This revealed enrichment of various metabolism related GO terms (Fig. 6).

### Genome wide DNA methylation

**Fig. 7:**
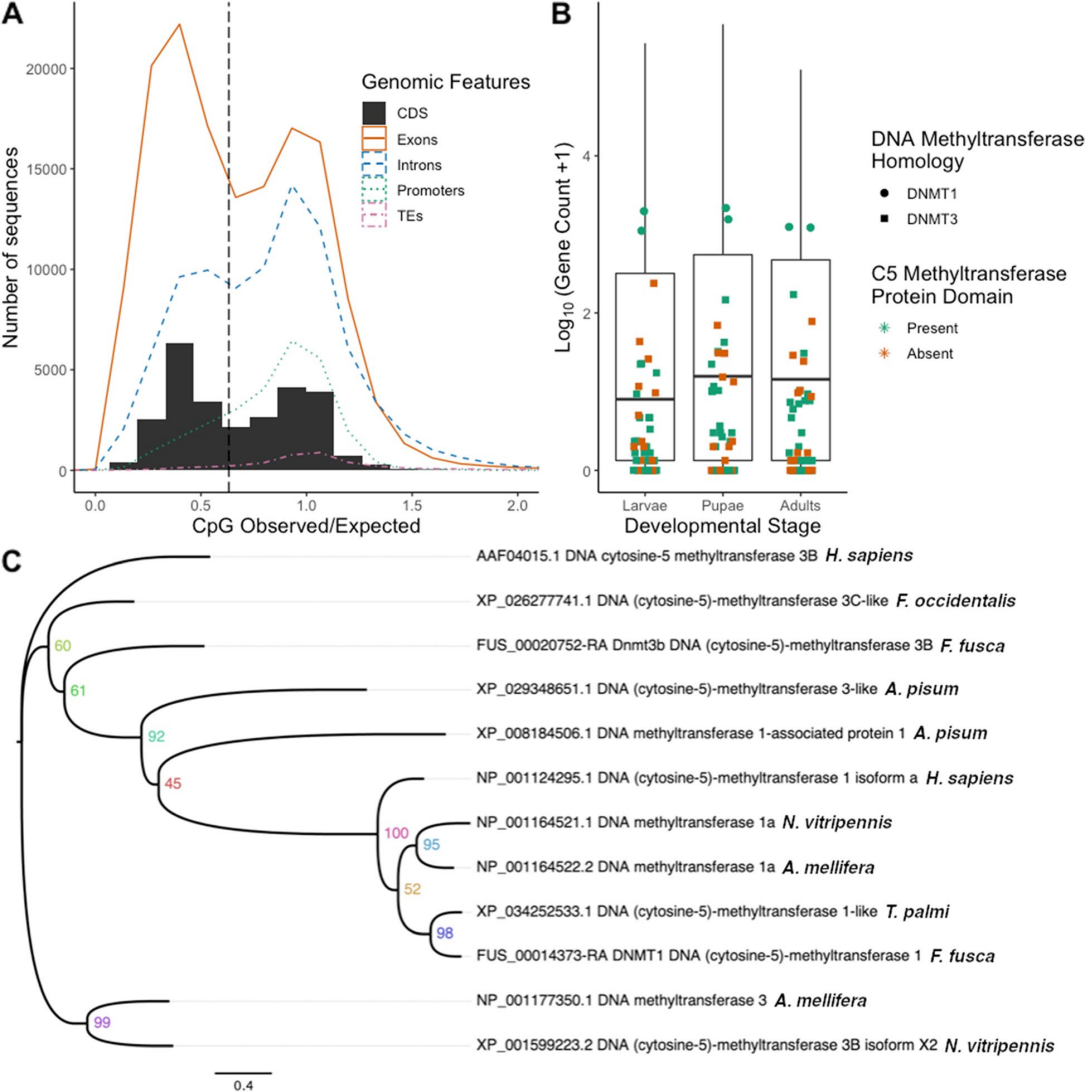
DNA methylation targets and molecular mediators. **A** CpGo/e of the *F. fusca* genomic features: exons, introns, and promoters and transposable elements (TEs) compared with the distribution of coding sequences (CDS). **B** DNA methyltransferase and DNMT-like gene expression level (gene count) in non-viruliferous individuals compared with the average expression level of all genes for each developmental stage. Individual points correspond to gene expression levels, whereas the boxplots are representative of the expression level distribution of the whole transcriptome per each developmental stage. The C5 methyltransferase domain presence or absence was classified by the InterPro Scan domain search (*n* = 52). **C** DNMT phylogenetic tree from *F. fusca*, *F. occidentalis*, *A. mellifera*, *N. vitripennis*, *A. pisum*, and *H. sapiens*. Numbers at branch nodes are confidence values based on 1000 bootstrap replications

Gene regulatory responses to orthotospovirus-infection may be influenced by epigenetic modifications leading to changes in accessibility for transcription factors. In insects, the levels of DNA methylation are generally low and concentrated within gene bodies [82, 83], where intragenic DNA methylation is hypothesized to influence alternative splicing and perhaps gene expression levels [84]. The mutational signatures of DNA methylation profiles from *F. fusca* and *F. occidentalis* were assessed by calculating normalized CpG content (CpGo/e), which can serve as a proxy measure for DNA methylation because CpG dinucleotides are the primary targets of DNA methylation in animals and methylated cytosines undergo deamination to thymine with high frequency [85–87]. The CpGo/e of the 26,416 and 23,148 coding sequences (CDS) in *F. fusca* and *F. occidentalis*, respectively, were found to have bimodal distributions with a mean CpGo/e of 0.68 for both (Additional file 1: Fig. S9A & S9B; Additional file 8: Table S18). The presence of many CpG-depleted coding sequences suggested that DNA methylation is targeted to those regions. Further investigation into the DNA methylation patterns of the genome was performed by examining CpGo/e distributions for various genomic features including exons, introns, promoter regions 1.5 kb upstream of the transcription start site (TSS), and transposable elements (TEs; Fig. 7A, Additional file 1: Fig. S10).

DNA methylation is controlled by the addition of a methyl group to cytosine by the maintenance DNA methyltransferase 1 (DNMT1) and the de novo DNA methyltransferase 3 (DNMT3) in insects and other eukaryotes [88]. The DNMT3s found in the *F. fusca* assembly were numerous but incomplete. The *F. fusca* DNMT3-like sequences collectively contained the expected domains, such as C5 methyltransferase (IPR001525) [89]. However, among the 50 DNMT3-like sequences identified, no single sequence contained all expected DNMT3 domains. The incomplete DNMT3s were mostly scattered across the genome assembly, with 27 having been found on separate contigs. Additionally, ten contigs were found to contain two DNMT3s and one contig contained three DNMT3s, which may represent tandem duplication. The expression levels of the *F. fusca* DNMT1 and DNMT3-like genes were compared against global gene expression levels for each *F. fusca* developmental stage (Fig. 7B) [90]. There was notable transcriptional activity of DNMT1 and many of the DNMT3-like sequences within *F. fusca* (Fig. 7B).

A phylogenetic tree of Blast-derived DNMTs was generated to investigate the relationship of a few confidently annotated *F. fusca* DNMT1 and DNMT3s to those from other insects and a vertebrate outgroup (Fig. 7C). DNMT1 hits were present in *F. fusca*, *T. palmi*, pea aphid (*Acyrthosiphon pisum* Harris), western honeybee (*Apis mellifera* Linnaeus), parasitoid wasp (*Nasonia vitripennis* Walker), and human (*Homo sapiens* Linnaeus), forming a clade in the phylogenetic tree consistent with expectations. However, the DNMT3 sequences did not resolve into a monophyletic group and were instead paraphyletic with respect to DNMT1 (Fig. 7C).

**Fig. 8:**
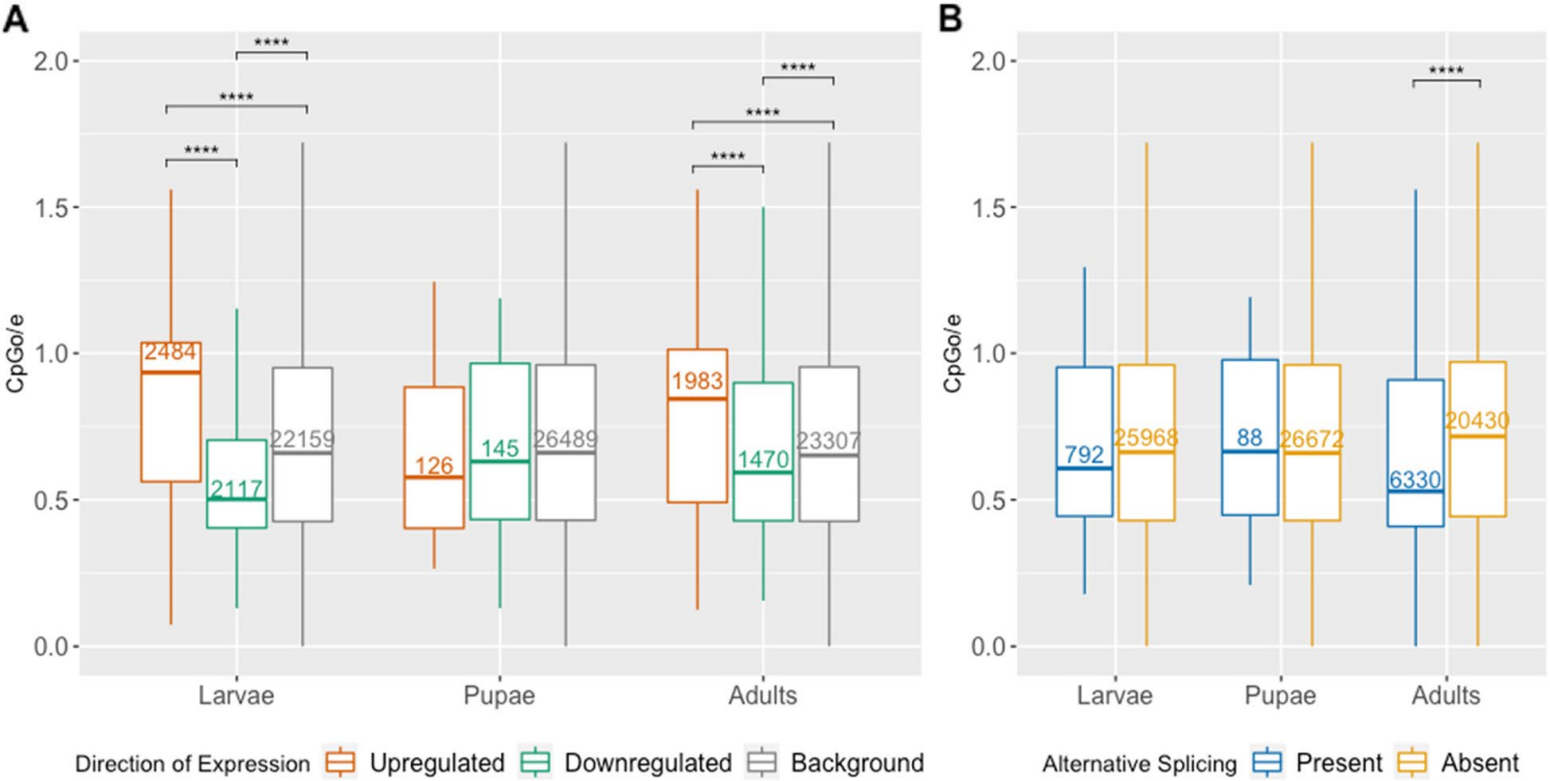
CpGo/e distributions of virus-responsive genes in *F. fusca*. **A** Differentially expressed genes and **B** alternatively spliced genes with differential exon usage across *F. fusca* developmental life stages. Lower CpGo/e reflects higher DNA methylation levels. Boxplot outliers not shown. Corresponding p-values: *p ≤ 0.05, **p ≤ 0.01, ***p ≤ 0.001, ****p ≤ 0.0001

Investigating the CpGo/e patterns for DEGs and AS genes in response to orthotospovirus-infection revealed that some classes of genes, as grouped according to their transcriptional attributes, were more likely to be targeted by DNA methylation than others (Fig. 8). Genes downregulated in response to orthotospovirus-infection exhibited significantly lower CpGo/e values than non-DEGs, suggesting these downregulated genes are preferentially targets of DNA methylation (Fig. 8A). In contrast, genes that were upregulated in response to orthotospovirus-infection exhibited significantly higher CpGo/e values than non-DEGs, suggesting these upregulated genes are less likely to be targets of DNA methylation than non-DEGs (Fig. 8A). Genes that were alternatively spliced in orthotospovirus-viruliferous versus non-viruliferous adults also were more likely to be targets of DNA methylation than genes that were not alternatively spliced (Fig. 8B).

## Discussion

This high-quality genome for the tobacco thrips is the third genome assembled in the order Thysanoptera after *F. occidentalis* [3] and *T. palmi* [2]. The quality of this genome can be attributed to PacBio sequencing, which reduced the total number of gaps relative to an assembly based on short-read sequences [3, 91, 92]. Despite the taxonomic closeness between the two *Frankliniella* species, their ecological interactions between hosts and viruses vary [12]. This study aimed at understanding those variations from physiological, evolutionary, and epigenetic perspectives.

Determination of dN/dS [93] was conducted for orthologous gene pairs between *F. fusca* and *F. occidentalis* to assess variation in selective pressures among genes. Genes with dN/dS > 1, consistent with positive selection, were identified among differentially regulated and alternative spliced genes following virus infection. ~ 97% of these genes were differentially regulated in *F. fusca* larvae and adults, but not in the least metabolically active pupal stage. Of the forty-seven 1:1 orthologous xenobiotic-metabolizing CYP enzymes [69, 94] identified, two exhibited dN/dS values consistent with positive selection. Acyl-CoA, which is also involved detoxification of xenobiotics in insects [65, 95, 96], also exhibited a signature of positive selection and was differentially expressed in larvae. Additional genes putatively subject to positive selection that were differentially regulated and/or alternatively spliced included heat shock proteins, which are known to inhibit virus infection in insects [72, 97]. Similarly, nicotinamide adenine dinucleotide (NADH) dehydrogenase exhibited a signature of positive selection and was differentially expressed in larvae and adults [98, 99]. The expression of these immunity-related genes may be influenced by epigenetic mechanisms that alter transcriptional activity in response to infection [100].

The CpGo/e of the *F. fusca* genome serves as a proxy to determine where DNA methylation has been present in the genome during recent evolutionary history [86, 101]. *F. fusca* exhibited a bimodal distribution of CpGo/e for exons, whereas other genomic features such as promoters and introns were found to have skewed/higher CpGo/e values, indicating less DNA methylation than exons, as is typical in insects [102]. A bimodal distribution showing high and low CpGo/e has been documented in the coding sequences of many insect species [88], including *F. occidentalis* [88], *A. mellifera* [86], two cricket species [59, 103], *A. pisum* [104], and two termite species [105].

When we assessed the signature of DNA methylation patterns for differentially expressed and alternatively spliced genes following virus infection, methylation was revealed to be preferentially targeted to genes alternatively spliced in adults and downregulated in adults and larvae following virus infection. Virus infection-induced changes in DNA methylation have been observed to occur among insects in the silkworm (*Bombyx mori* Linnaeus) following infection by *B. mori* cytoplasmic polyhedrosis virus (BmCPV) [106]. Methylation patterns in eukaryotes are established and maintained by DNA methyltransferases DNMT3 and DNMT1 respectively [107, 108]. While the function of the multiple DNMT3s we observed in *F. fusca* has yet to be resolved, one possible explanation for the high copy number is that some of these DNMT3s have undergone subfunctionalization, in which two or more copies of a duplicated genes divide the function of the ancestral gene [109, 110]. Another possibility is neofunctionalization of DNMT3 alleles [111]. It has been suggested that some DNMTs in other arthropod species may have varying biological processes [108, 112, 113]. Further exploration into the DNMTs in *F. fusca* may resolve functionality of these genes.

## Conclusions

This study generated a genomic resource for *F. fusca* using long-read sequence data. The genome assembly allowed for investigation into the evolutionary history of *F. fusca* in more depth than was possible with a transcriptome. The assembly allowed for the reanalyzing of previously published RNA-seq data [32] for differential expression and novel analysis of alternative splicing events following orthotospovirus-infection. By comparing the sequence divergence of protein-coding genes in *F. fusca* with orthologs in *F. occidentalis*, we revealed many genes that may be evolving under positive selective pressure relating to orthotospovirus-infection. Within the genus *Orthotospovirus*, TSWV is one of the most studied thrips-transmitted viruses, which has contributed broadly to our understanding of virus-insect interactions [114].

The CpGo/e patterns of differentially expressed and alternatively spliced genes indicated different patterns of potential methylation events in relation to orthotospovirus-infection. Additionally, high counts of incomplete DNA methyltransferases were found within the genome, consistent with sub- or neo-functionalization. This genomic resource should facilitate further investigation into the potential roles of DNA methylation in thrips gene regulation and virus transmission. The addition of more high-quality thrips genomes will broaden these inferences.

## Materials and methods

### Sampling, extraction, and sequencing

One thousand male *F. fusca* heads from an 11^th^ generation inbred line were used for DNA extraction. Samples were pooled to obtain enough material for high quality library construction. Heads were chosen to reduce potential sources of contamination. The Qiagen DNeasy Blood & Tissue kit was used for extraction with half the recommended volumes for each reagent and 50 μL elution. An Illumina sequencing library was constructed, and samples were sequenced on a HiSeq 2000 used with 150PE reads at the University of Maryland Genomic Facility. PacBio library preparation was performed with the Sequel-2 chemistry kit and sequenced on two single-molecule real-time (SMRT) cells, also at the University of Maryland Genomic Facility. Voucher specimens were retained and stored in 95% alcohol at -20C at North Carolina State University.

### Genome assembly and quality control

The assembly was performed with the use of the program Flye v2.6 [115, 116], which ran for two iterations with the expected haploid genome size being 400 Mb [117]. This unphased assembly had Illumina sequence data mapped to it using Minimap2 v2.17 [118] and Samtools v1.10 [119, 120]. Error correction was performed with Pilon v1.10 [121] using the mapped Illumina reads. Assembly quality was checked with Quality Assessment Tool for Genome Assemblies (QUAST) v5.0.2 [122] and BUSCO v4.0.5 [34]. Assembly fragments larger than 2.5 Mb were extracted with Seqtk (https://github.com/lh3/seqtk). The genome assembly was visualized using Bandage v0.8.1 [123] and Circos v0.69 [124]. The BUSCO scores were searched against the Insecta odb10 with the species set to the default of “Fly”. The BUSCO scores were checked against the 1,367 core genes that exist in the Insecta odb10. The BUSCO Insecta odb10 is comprised of core genes from 75 insect species across 14 orders, which includes Thysanoptera as represented by *F. occidentalis*. Screening was performed with recommended VecScreen [125] parameters being “-task blastn -reward 1 -penalty -5 -gapopen 3 -gapextend 3 -dust yes -soft_masking true -evalue 700 -searchsp 1,750,000,000,000” to remove any potential contamination. The quality of the gene models were assessed using BUSCO v4 and the lineage Insecta odb10 ortholog set with 1,367 core genes [34]. Counting of k-mers was conducted using the tool JELLYFISH for k-mers of length 19, 21, 23, 25, 27, 31, and 100 (Additional file 1: Fig. S11A) [126].

### Genome annotation

To run the annotation steps efficiently, the main assembly file was split up into smaller subfiles using BBMap v38.73 parition.sh [127]. Each subfile contained about 5% (or 72) of the contigs from the main file. The tool MAKER v3.01.02-beta [35] was used and run through four rounds to refine the annotation quality. The first round of MAKER included external evidence from a *F. fusca* and *F. tritici* transcriptome, *F. occidentalis* transcriptome and proteome, and additional proteomes from UniProt: fruit fly (*Drosophila melanogaster* Meigen) (UP000000803), *A. pisum* (UP000007819), red flour beetle (*Tribolium castaneum* Herbst) (UP000007266), body louse (*Pediculus humanus subsp. Corporis* Linneaus) (UP000009046), and the Nevada termite (*Zootermopsis nevadensis* Hagen) (UP000027135). Subsequent rounds of MAKER required training with SNAP-Zoe v2006-07–28 (http://www.hiv.lanl.gov) [128] and AUGUSTUS v3.2.3 [129] with the output from the previous MAKER run (AUGUSTUS also requires BEDTools v2.29.2 for proper formatting). The total quality of each round of MAKER was assessed using annotation edit distance (AED) scores, which is a metric describing how well genome annotations are supported by evidence such as the sequence homology and expressed sequence tags (ESTs) [130]. Repeat masking was handled internally within MAKER using RepeatMasker v4.0.7 (A.F.A. Smit, R. Hubley & P. Green RepeatMasker at http://repeatmasker.org). All rounds of MAKER were set to generate a single annotation for each gene using the default parameter (alt_splice = 0). The functional annotations were performed using the OmicsBox v1.4.11 toolkit [131]. Web Gene Ontology Annotation Plot (WEGO) v2 was used to visualize and determine significant enrichment of GO terms [132, 133].

### Orthologous gene detection

OrthoFinder v2.3.7 [134, 135] was used to construct a species tree based on several insect proteomes, using the following parameters: gene tree inference (-M) msa, multiple sequence alignment (-A) mafft [136], and tree inference program (-T) raxml [137]. This tree was generated from OrthoFinder2, which internally uses the programs STRIDE and STAG for comparing proteins and generating orthogroups [138]. The tree was viewed using the tool FigTree v1.4.4 (https://github.com/rambaut/figtree/) from the output from IQ-TREE v1.6.12 after 1000 iterations to calculate bootstrap values [139–141]. Additional gene family comparisons were made with OrthoVenn2 using a cutoff of e^−10^ [60]. Eggnog-mapper v1.0.3 was used to assign GO terms using the eggNOG database [36, 142]. This was accomplished using default parameters and checking across all available species and genes in the database.

Gene counts for detoxification and virus responsive genes were determined by searching the InterPro database [143]. Genes containing the following domains were considered: heat shock protein 70 family (IPR013126), heat shock protein Hsp90, N-terminal (IPR020575), acyl-CoA desaturase (IPR015876), mitogen-activated protein (MAP) kinase, JNK (IPR008351), dicer dimerisation domain (IPR005034), chytochrome P450 (IPR001128), ABC transporter-like, ATP-binding domain (IPR003439), glutathione S-transferase, N-terminal (IPR004045), phospholipase/ carboxylesterase/ thioesterase (IPR003140), aminopeptidase N-like (IPR033581), peroxiredoxin, C-terminal (IPR019479), peptidoglycan recognition protein (IPR015510), amino acid transporter, transmembrane domain (IPR013057), serine proteases, trypsin domain (IPR001254), and 40S ribosomal protein (IPR032277).

### Differentially expressed and alternatively spliced genes

The RNA-Seq data comes from the NCBI sequence read archives (SRA): SRX2788009, SRX2788011, SRX2788013, SRX2788010, SRX2788012, and SRX2788014 [57]. RNA-Seq by Expectation–Maximization (RSEM) v1.3.3 and Bowtie2 v2.4.1 were used for mapping to the reference gene models and determination of gene counts with default parameters (~ 42–65% alignment; Additional file 1: Table S19) [144, 145]. DESeq2 v3.13 was used for differential expression analysis [146]. STAR aligner v2.7.9 was used with default parameters to map reads to the genome (~ 87–94% alignment; Additional file 1: Table S20) [147]. SAMtools v1.10 was used to sort the alignments [119]. DEXSeq was used with default parameters to calculate the amount of alternative splicing of exons [148].

### Protein evolution rate

The 1:1 orthologs detected from Orthofinder2 were used for dN/dS calculations. Pal2Nal v14 [149] and PAML v4.9 [150] were used to calculate the respective dN/dS values. Using custom R script, genes were partitioned into significantly and non-significantly expressed genes for each stage.

### GO term enrichment

The Bioconductor package topGO (https://bioconductor.org/packages/release/bioc/html/topGO.html) was used to determine the significantly enriched GO terms from genes of interest under the DEG, AS genes, and the intersection between DEGs and AS genes. Revigo and rrvgo (https://bioconductor.org/packages/release/bioc/html/rrvgo.html), with respect to the *D. melanogaster* ortholog database, were used downstream to visualize the GO term enrichment [151].

### DNA methylation targeting of genomic elements

Determination of the observed to expected CpG content in the coding regions of the genome was performed using CpG.pl (https://github.com/swebb1/cpg_tools) using the default parameters. GffRead v0.9.12 was used to extract the CDS [152]. BEDTools v2.26.0 was used to extract the exons, introns, and promoter regions [153]. EDTA v1.9.9 was used to annotate the TEs using default parameters (Additional file 1: Fig. S11B) [154–161]. Promoter regions were parsed out using (https://github.com/milesroberts-123/extract-promoter-sequences), with the parameters upstream (-u) 1500 and downstream (-d) 0 from the transcription start site. Characterization of the DNMTs in *F. fusca* was performed using the OmicsBox v1.4.11 toolkit [131], which includes Blast2GO [162] and Pfam database search [41]. The resulting OmicsBox annotations were compared with MAKER v3.01.02-beta [35] annotations, which were assigned by InterProScan [163]. The DNMT tree was built from an InterProScan of *A. mellifera* DNMT domain BlastP [164] with default parameters against the following species: *F. fusca*, *F. occidentalis*, *A. mellifera*, *N. vitripennis*, *A. pisum*, and *H. sapiens*. The tree was viewed using the tool FigTree v1.4.4 (https://github.com/rambaut/figtree/) from the output from IQ-TREE v1.6.12 after 1000 iterations over the mafft multiple alignments [136] to calculate bootstrap values [139–141].

## Supplementary Information

**Additional file 1.** 

**Additional file 2.** 

**Additional file 3.** 

**Additional file 4.** 

**Additional file 5.** 

**Additional file 6.** 

**Additional file 7.** 

**Additional file 8.**

## Data Availability

The data for this article can be found in the NCBI GenBank repository at https://www.ncbi.nlm.nih.gov/ under the BioProject PRJNA742006. Raw sequence data for the BioSample SAMN19606609 are deposited in the SRA accessions: SRR15147323-SRR15147327. The genome and transcriptome shotgun assembly (TSA) submission accessions are JAHWGI000000000 and GJHY00000000, respectively. Supplementary information and captions can be found in Additional file 1: Figures S1-S11 and Tables S1-S20. Respective supplementary tables can be found in Additional file 2: Table S2, Additional file 3: Tables S4 & S5, Additional file 4: Tables S6 & S7, Additional file 5: Tables S8-S11, Additional file 6: Tables S12-S14, Additional file 7: Tables S15-S17, and Additional file 8: Table S18.

## Abbreviations

TSWV: Tomato spotted wilt orthotospovirus
CNV: Copy number variation
ABC: ATP-binding cassette
CE: Carboxylesterase
CYP: Cytochrome P450 monooxygenase
HSP: Heat shock protein
MAP: Mitogen-activated protein
JNK: Jun N-terminal kinase
PGRP: Peptidoglycan recognition protein
dN/dS: Non-synonymous substitutions and synonymous substitutions ratio
CpGo/e: CpG observed/expected
TE: Transposable element
DNMT: DNA methyltransferase
BUSCO: Benchmarking Universal Single-Copy Orthologs
KEGG: Kyoto Encyclopedia of Genes and Genomes
GO: Gene ontology
DEG: Differentially expressed genes
AS: Alternatively spliced
CDS: Coding sequence
UTR: Untranslated region
TSS: Transcription start site
NADH: Nicotinamide adenine dinucleotide
SMRT: Single-molecule real-time
QUAST: Quality Assessment Tool for Genome Assemblies
WEGO: Web Gene Ontology Annotation Plot
RBH: Reciprocal best hits
NCBI: National Center for Biotechnology Information
SRA: Sequence read archives
RSEM: RNA-Seq by Expectation–Maximization
TSA: Transcriptome shotgun assembly
