# Supplementary material for: Pest status, molecular evolution, and epigenetic factors derived from the genome assembly of Frankliniella fusca, a thysanopteran phytovirus vector
Source: BMC Genomics. 2023 Jun 22;24:343. doi: 10.1186/s12864-023-09375-5 (PMC10286387; doi:10.1186/s12864-023-09375-5)
Supplement: Supplementary file 1 — Additional file 1. [file 12864_2023_9375_MOESM1_ESM.doc]

**Supplementary Figures and Tables for**

Pest status, molecular evolution, and epigenetic factors derived from the genome assembly of *Frankliniella fusca*, a thysanopteran phytovirus vector

Michael A. Catto, Paul E. Labadie, Alana L. Jacobson, George G. Kennedy, Rajagopalbabu Srinivasan, and Brendan G. Hunt

**Correspondence to:** [huntbg@uga.edu](mailto:huntbg@uga.edu) (B.G.H); [babusri@uga.edu](mailto:babusri@uga.edu) (R.S)

**
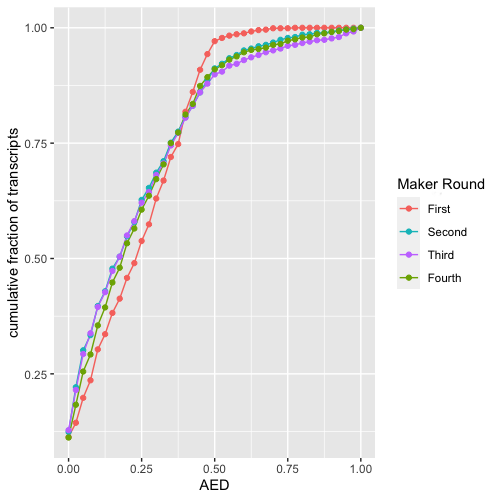
**

**Fig. S1.** **AED score distribution of all rounds of MAKER.** In the final round, ~60% of all transcripts fall below an AED score of 0.25. Each round improved the overall quality of the annotations, with the final round being the highest quality.


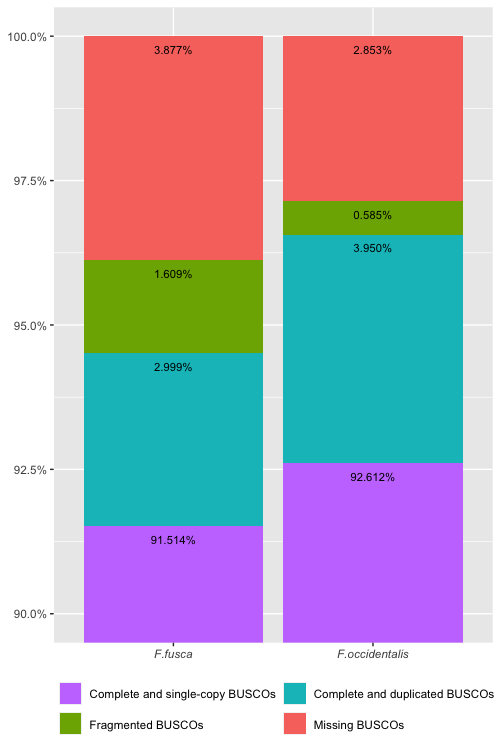


**Fig. S2. Final annotations BUSCO scores for *F. fusca* and *F. occidentalis* against the Insecta odb10 lineage. (n = 1,367).**


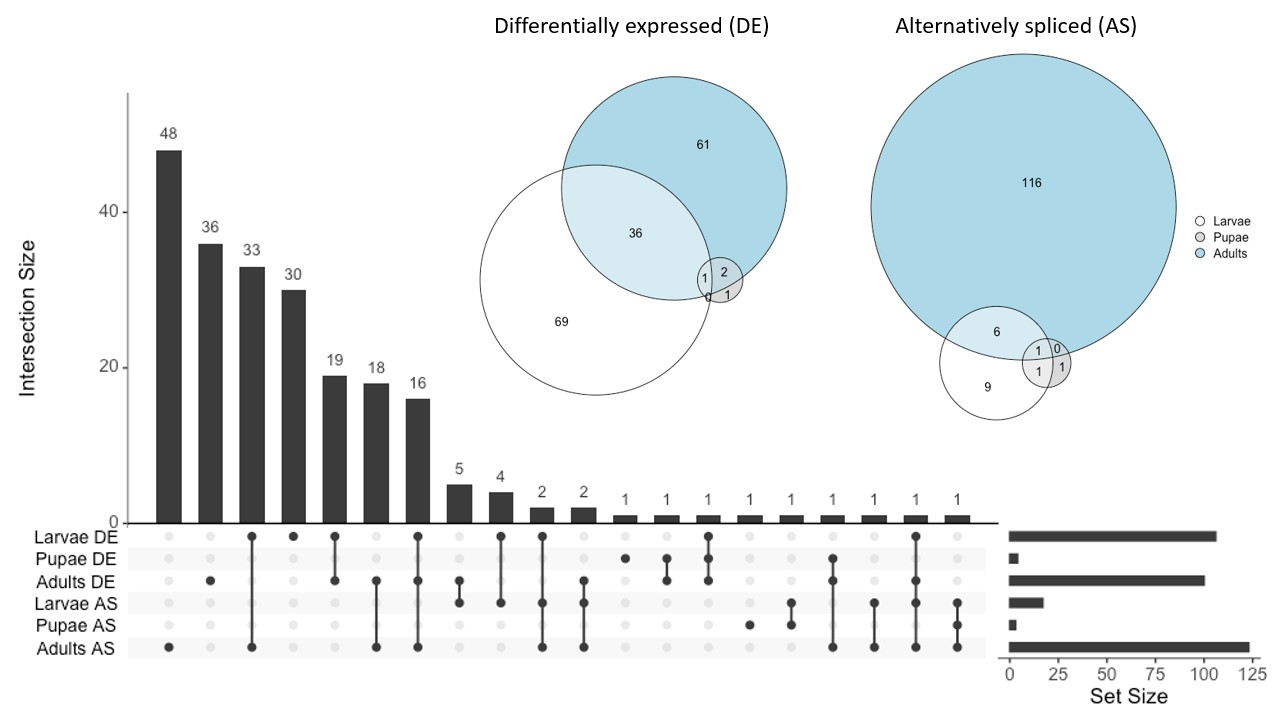
**Fig. S3. Overlap across *F. fusca* developmental stages for virus-responsive genes.** Overlap between developmental stages of differentially expressed (DE) genes (n = 170) and alternatively spliced (AS) genes (n = 134). Upset plot (below) indicating overlap of DE and AS genes with dN/dS > 1.


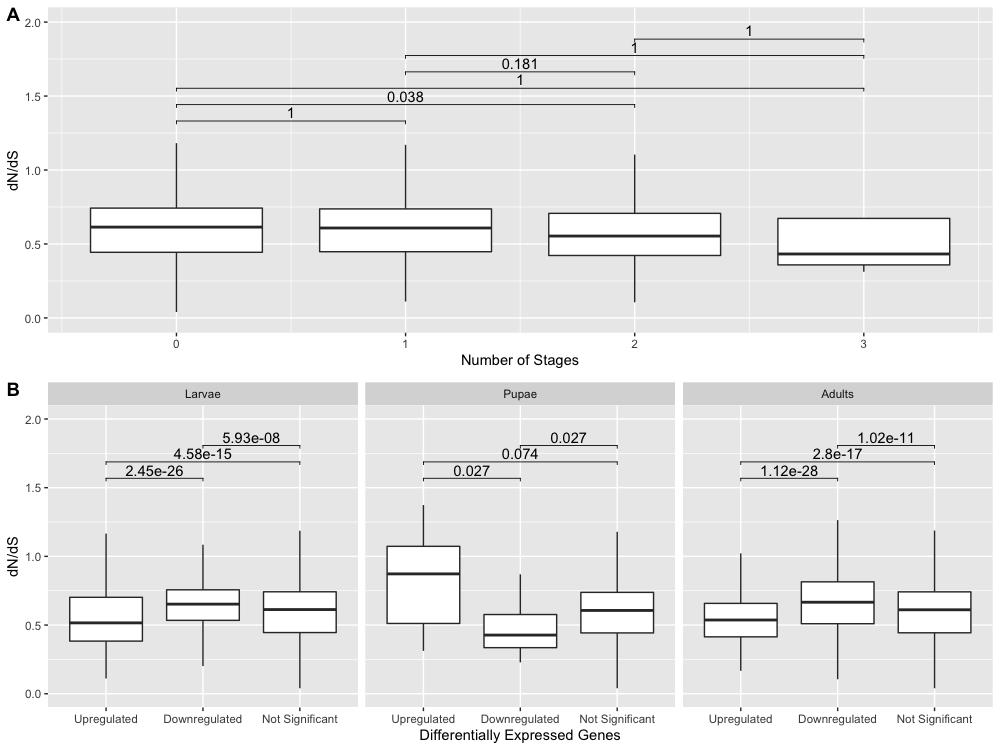


**Fig. S4. Comparison of rates of non-synonymous to synonymous mutations (dN/dS) of differentially expressed genes between developmental stages.** (**A**) Comparisons of dN/dS between differentially expressed genes within 0 (non-significant genes), 1, 2, or 3 developmental groups (n = 7,817). (**B**) dN/dS of differentially expressed genes considering the direction of expression. Outliers were excluded from the visualization.


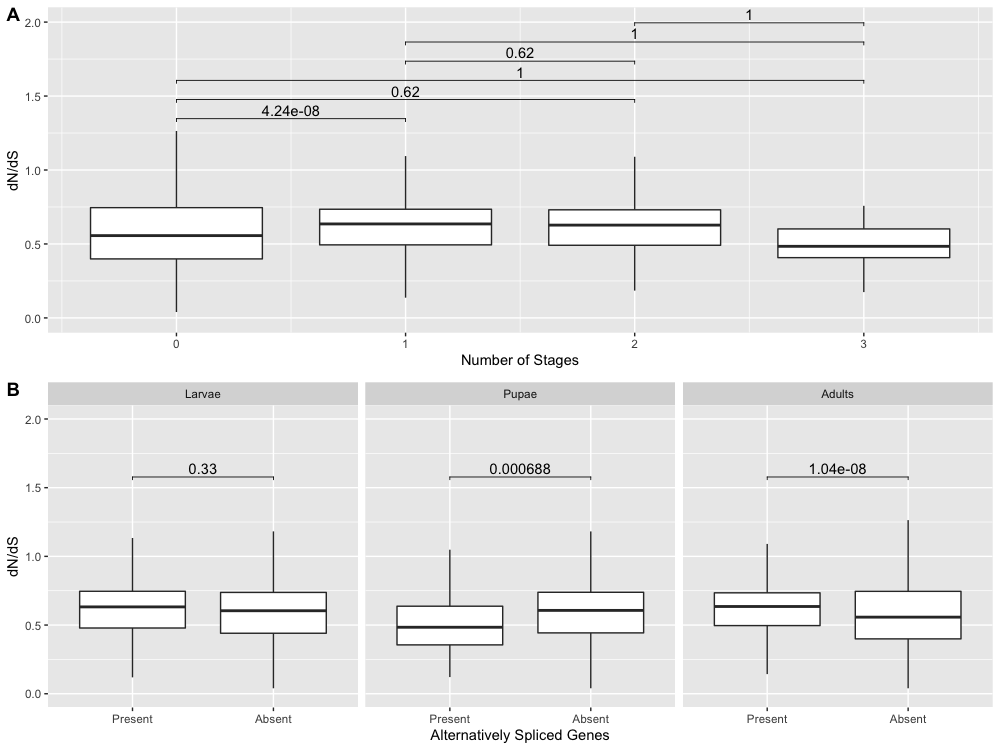


**Fig. S5.** **Comparison of rates of non-synonymous to synonymous mutations (dN/dS) of alternatively spliced genes between developmental stages.** (**A**) Comparisons of dN/dS between alternatively spliced genes within 0 (non-significant genes), 1, 2, or 3 developmental groups (n = 7,817). (**B**) dN/dS of alternatively spliced genes with respect to presence or absence of differential exon usage. Outliers were excluded from the visualization.


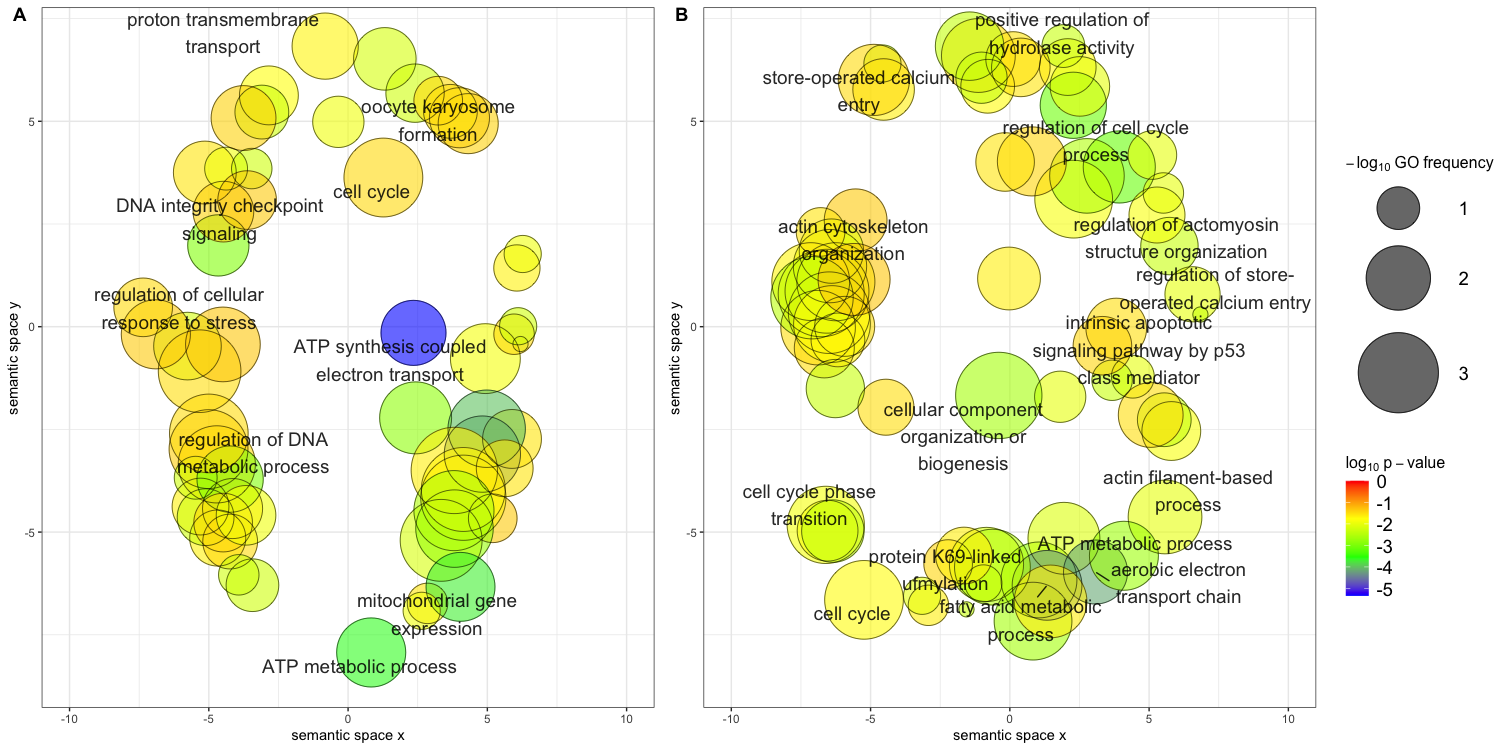


**Fig. S6. Biological process GO term ratios based on semantic term clustering with dN/dS > 1.** GO term enrichment (p < 0.05) for genes with dN/dS > 1 within the biological processes (BP) category of GO terms for (**A**) differentially expressed genes and (**B**) alternatively spliced genes. Bubble color indicates p-value and bubble size represents GO term frequency.


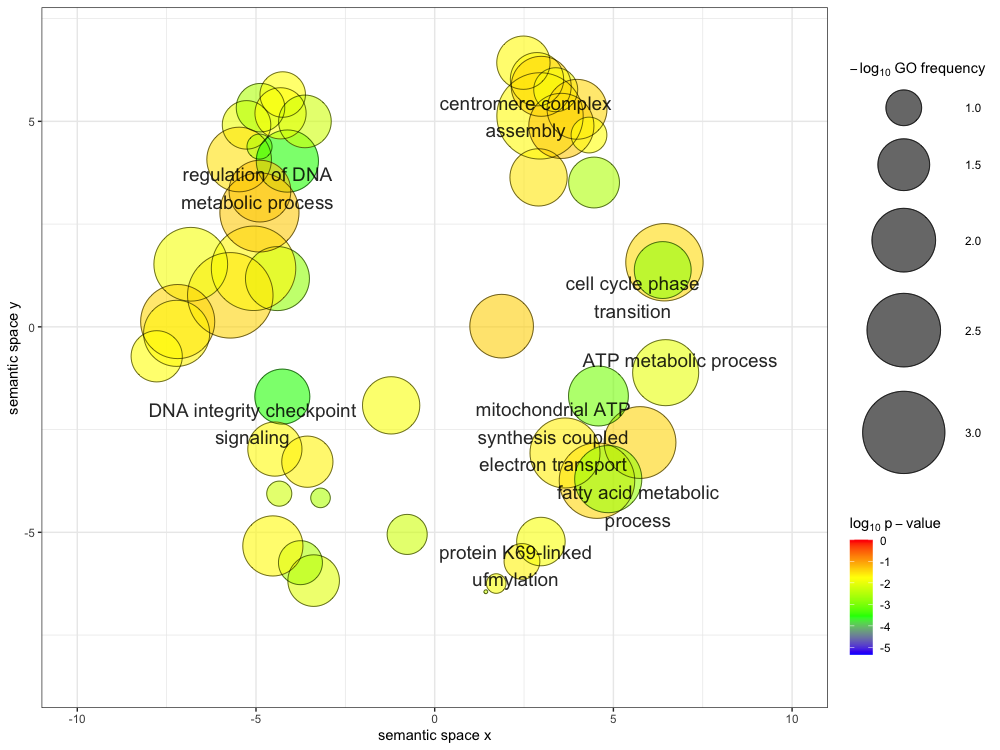


**Fig. S7. Biological process GO term ratios based on semantic term clustering with dN/dS > 1.** GO term enrichment (p < 0.05) for genes with dN/dS > 1 within the biological processes (BP) category of GO terms, representing the intersection between differentially expressed and alternatively spliced genes. Bubble color indicates p-value and bubble size represents GO term frequency.


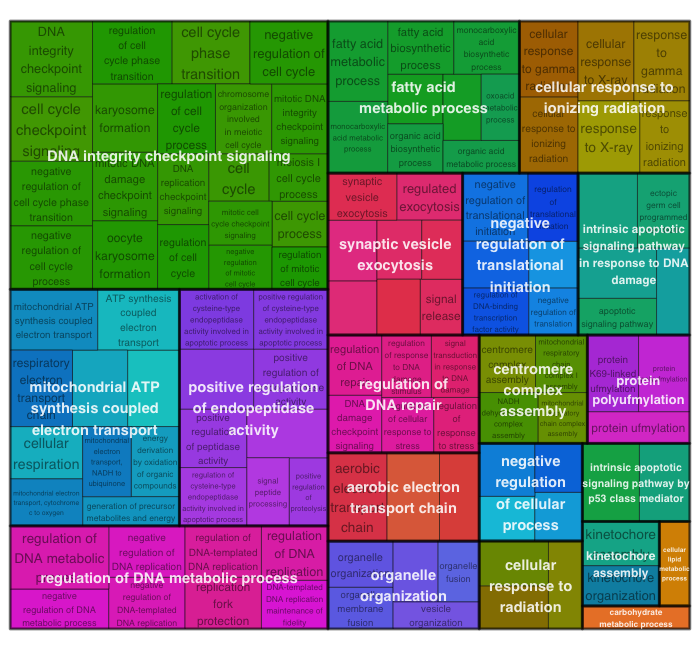


**Fig. S8.** **Tree map GO term enrichment for virus-responsive genes with dN/dS > 1.** GO term enrichment (p < 0.05) for genes with dN/dS > 1 within the biological processes (BP) category of GO terms, representing the intersection between differentially expressed and alternatively spliced genes. Each individual box represents a single GO term, with one representative term per supercluster. The size of each box relates to the relative frequency of the respective GO term.


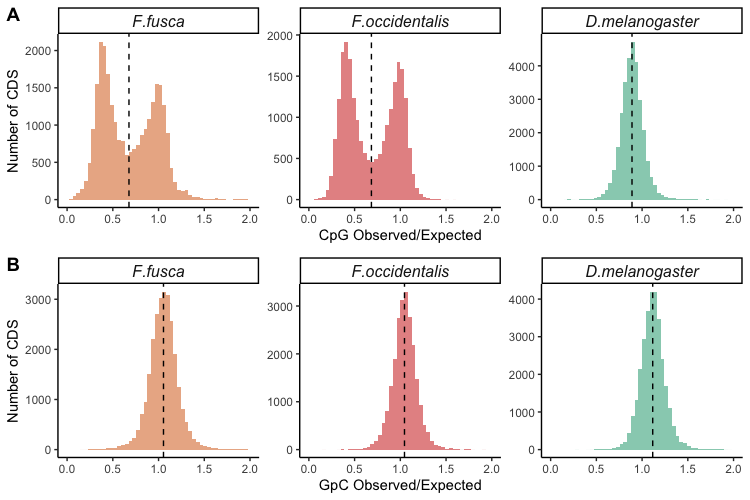


**Fig. S9.** **Epigenome profiles of *Frankliniella fusca*, *Frankliniella occidentalis*, and *Drosophila melanogaster*.** (**A**) The CpGo/e distribution is bimodal in both thrips’ species, compared to the unmethylated fruit fly genome. (**B**) The GpCo/e distribution shows the absence of any strong signal outside of random chance.


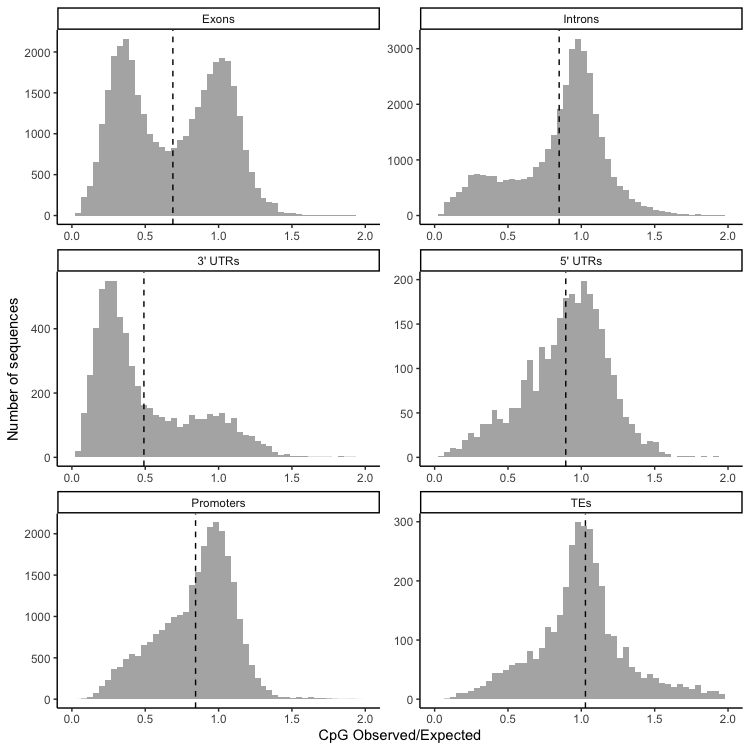


**Fig. S10. CpG content (CpGo/e) distributions.** Introns (n = 36,957), exons (n = 37,742), 3’ untranslated regions (n = 6,288), and 5’ untranslated regions (n = 2,896) filtered at < 300bp to reduce noise. Promoter region (n = 26,732). Transposable elements (n = 3,709).


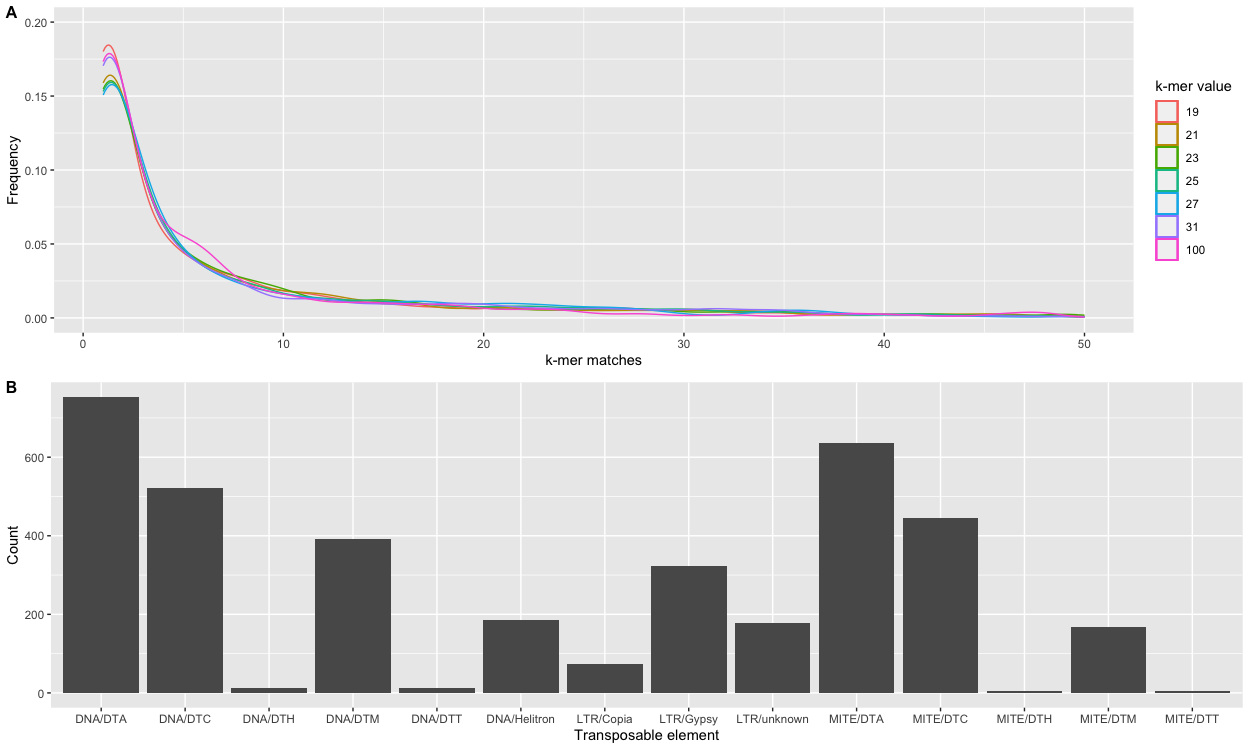


**Fig. S11. k-mer frequency and transposable element counts within the genome assembly.** (**A**) Various k-mer distributions across the genome assembly assessed with JELLYFISH. (**B**) Transposable element (TE) annotation counts found within the genome assembly using the EDTA tool.

**Table S1. Number of gene models produced from each round of MAKER.** The average gene length increased with each round, showing improvements to the overall quality of the annotated genome.

|  | Round 1 | Round 2 | Round 3 | Round 4 |
| --- | --- | --- | --- | --- |
| Gene Models | 24,415 | 27,404 | 26,808 | 26,761 |
| Average Gene Length | 3,373.38 | 4,064.9 | 4,311 | 4,340.52 |

**Table S2. Genome annotations derived from the eggNOG database.** 17,389 proteins were assigned functional annotation. **See Additional file 2.**

**Table S3. Orthogroups shared between *F. fusca* and *F. occidentalis.*** Comparison statistics are derived from the entire proteomes of both species using Orthofinder.

|  | *Frankliniella fusca* | *Frankliniella occidentalis* |
| --- | --- | --- |
| Number of genes | 26,761 | 23,356 |
| Number of genes in orthogroups | 23,358 | 19,846 |
| Number of unassigned genes | 3,403 | 3,510 |
| Percentage of genes in orthogroups | 87.3 | 85.0 |
| Percentage of unassigned genes | 12.7 | 15.0 |
| Number of orthogroups containing species | 12,596 | 12,197 |
| Percentage of orthogroups containing species | 94.5 | 91.5 |
| Number of species-specific orthogroups | 1,127 | 728 |
| Number of genes in species-specific orthogroups | 7,752 | 2,684 |
| Percentage of genes in species-specific orthogroups | 29.0 | 11.5 |

**Table S4. 7,912 one-to-one orthologs list from *F. fusca* and *F. occidentalis*. See Additional file 3.**

**Table S5. dN/dS and alignment statistics of one-to-one orthologs from *F. fusca* and *F. occidentalis*. See Additional file 3.**

**Table S6. Total protein domain counts of relevant detoxification molecules within *F. fusca*, *F. occidentalis*, and orthologs of *F. fusca* and *F. occidentalis*. See Additional file 4.**

**Table S7. Individual protein domain counts per gene within *F. fusca*, *F. occidentalis*, and orthologs of *F. fusca* and *F. occidentalis*. See Additional file 4.**

**Table S8. Expression differences from the comparison of TSWV-infected to non-infected developmental stages of *F. fusca*.** Genes with a dN/dS > 1 were selected from the one-to-one orthologs between *F. fusca* and *F. occidentalis*. Significance values are denoted by * = p ≤ 0.05, ** = p ≤ 0.01, and *** = p ≤ 0.001. **See Additional file 5.**

**Table S9. Counts of alternative splicing events from the comparison of TSWV-infected to non-infected developmental stages of *F. fusca*.** Genes with a dN/dS > 1 were selected from the one-to-one orthologs between *F. fusca* and *F. occidentalis*. **See Additional file 5.**

**Table S10. Expression differences and counts alternative splicing events from the comparison of TSWV-infected to non-infected developmental stages of *F. fusca*.** Genes with a dN/dS > 1 were selected from the one-to-one orthologs between *F. fusca* and *F. occidentalis*. Significance values are denoted by * = p ≤ 0.05, ** = p ≤ 0.01, and *** = p ≤ 0.001. **See Additional file 5.**

**Table S11. Comparison of significance between differentially expressed genes (DEGs) and nonDEGs, alternatively spliced (AS) genes and nonAS genes, and genes that were both differentially expressed and contained alternative splicing events from the comparison of TSWV-infected to non-infected *F. fusca* samples.** Genes with a dN/dS > 1 and dN/dS < 1 were compared within each respective group. **See Additional file 5.**

**Table S12. DEGs of the larval developmental stage from the comparison of TSWV-infected to non-infected *F. fusca* samples. See Additional file 6.**

**Table S13. DEGs of the pupal developmental stage from the comparison of TSWV-infected to non-infected *F. fusca* samples. See Additional file 6.**

**Table S14. DEGs of the adult developmental stage from the comparison of TSWV-infected to non-infected *F. fusca* samples. See Additional file 6.**

**Table S15. Differential exon usage (AS events) of the larval developmental stage from the comparison of TSWV-infected to non-infected *F. fusca* samples. See Additional file 7.**

**Table S16. Differential exon usage (AS events) of the pupal developmental stage from the comparison of TSWV-infected to non-infected *F. fusca* samples. See Additional file 7.**

**Table S17. Differential exon usage (AS events) of the adult developmental stage from the comparison of TSWV-infected to non-infected *F. fusca* samples. See Additional file 7.**

**Table S18. CpGo/e and associated statistics derived from *F. fusca* gene models. See Additional file 8.**

**Table S19. Bowtie2 mapping statistics for RNA-Seq data. Reads aligned to the genome 0, 1, or >1 time(s). Overall alignment rate includes 1 or >1 time(s).**

| Sample ID | aligned concordantly 0 times | aligned concordantly exactly 1 time | aligned concordantly >1 times | overall alignment rate |
| --- | --- | --- | --- | --- |
| HA1_AGTCAA_L008_R1_001 | 7534906 (47.09%) | 7573841 (47.34%) | 891253 (5.57%) | 52.91% |
| HA1_AGTCAA_L008_R1_002 | 7576571 (47.35%) | 7544873 (47.16%) | 878556 (5.49%) | 52.65% |
| HA1_AGTCAA_L008_R1_003 | 2066513 (47.63%) | 2037355 (46.96%) | 234420 (5.40%) | 52.37% |
| HA2_AGTTCC_L008_R1_001 | 9072125 (56.70%) | 6181424 (38.63%) | 746451 (4.67%) | 43.30% |
| HA2_AGTTCC_L008_R1_002 | 9107382 (56.92%) | 6155913 (38.47%) | 736705 (4.60%) | 43.08% |
| HA2_AGTTCC_L008_R1_003 | 1832221 (57.73%) | 1201911 (37.87%) | 139514 (4.40%) | 42.27% |
| HA3_ATGTCA_L008_R1_001 | 7900549 (49.38%) | 7057194 (44.11%) | 1042257 (6.51%) | 50.62% |
| HA3_ATGTCA_L008_R1_002 | 5835413 (49.67%) | 5159219 (43.91%) | 753670 (6.42%) | 50.33% |
| HL1_CGATGT_L006_R1_001 | 6535157 (40.84%) | 8400786 (52.50%) | 1064057 (6.65%) | 59.16% |
| HL1_CGATGT_L006_R1_002 | 6452104 (41.29%) | 8153227 (52.17%) | 1022221 (6.54%) | 58.71% |
| HL2_TGACCA_L006_R1_001 | 6420022 (40.13%) | 8698370 (54.36%) | 881608 (5.51%) | 59.87% |
| HL2_TGACCA_L006_R1_002 | 6496324 (40.60%) | 8635612 (53.97%) | 868064 (5.43%) | 59.40% |
| HL2_TGACCA_L006_R1_003 | 366233 (41.65%) | 467301 (53.15%) | 45751 (5.20%) | 58.35% |
| HL3_ACAGTG_L006_R1_001 | 6184645 (38.65%) | 8847744 (55.30%) | 967611 (6.05%) | 61.35% |
| HL3_ACAGTG_L006_R1_002 | 6270196 (39.19%) | 8780361 (54.88%) | 949443 (5.93%) | 60.81% |
| HL3_ACAGTG_L006_R1_003 | 1929391 (38.72%) | 2755384 (55.30%) | 297696 (5.97%) | 61.28% |
| HP1_GCCAAT_L007_R1_001 | 6629195 (41.43%) | 8925975 (55.79%) | 444830 (2.78%) | 58.57% |
| HP1_GCCAAT_L007_R1_002 | 6671104 (41.69%) | 8888383 (55.55%) | 440513 (2.75%) | 58.31% |
| HP1_GCCAAT_L007_R1_003 | 3095325 (41.97%) | 4078997 (55.31%) | 201134 (2.73%) | 58.03% |
| HP2_CAGATC_L007_R1_001 | 6638053 (41.49%) | 8881160 (55.51%) | 480787 (3.00%) | 58.51% |
| HP2_CAGATC_L007_R1_002 | 6683017 (41.77%) | 8840735 (55.25%) | 476248 (2.98%) | 58.23% |
| HP2_CAGATC_L007_R1_003 | 623511 (42.87%) | 789357 (54.28%) | 41474 (2.85%) | 57.13% |
| HP3_CTTGTA_L007_R1_001 | 6496151 (40.60%) | 8922169 (55.76%) | 581680 (3.64%) | 59.40% |
| HP3_CTTGTA_L007_R1_002 | 6537447 (40.86%) | 8887321 (55.55%) | 575232 (3.60%) | 59.14% |
| HP3_CTTGTA_L007_R1_003 | 751660 (41.73%) | 987945 (54.85%) | 61488 (3.41%) | 58.27% |
| IA1_GATCAG_L008_R1_001 | 6401160 (40.01%) | 8750934 (54.69%) | 847906 (5.30%) | 59.99% |
| IA1_GATCAG_L008_R1_002 | 5158599 (40.09%) | 7034411 (54.66%) | 676119 (5.25%) | 59.91% |
| IA2_TAGCTT_L008_R1_001 | 6464882 (40.41%) | 8649308 (54.06%) | 885810 (5.54%) | 59.59% |
| IA2_TAGCTT_L008_R1_002 | 6489746 (40.56%) | 8631073 (53.94%) | 879181 (5.49%) | 59.44% |
| IA2_TAGCTT_L008_R1_003 | 1168053 (41.40%) | 1503829 (53.30%) | 149534 (5.30%) | 58.60% |
| IA3_GGCTAC_L008_R1_001 | 7416085 (46.35%) | 7691674 (48.07%) | 892241 (5.58%) | 53.65% |
| IA3_GGCTAC_L008_R1_002 | 7428233 (46.43%) | 7685161 (48.03%) | 886606 (5.54%) | 53.57% |
| IA3_GGCTAC_L008_R1_003 | 3072328 (46.68%) | 3148460 (47.84%) | 360973 (5.48%) | 53.32% |
| IL1_CCGTCC_L006_R1_001 | 5496360 (34.35%) | 8653987 (54.09%) | 1849653 (11.56%) | 65.65% |
| IL1_CCGTCC_L006_R1_002 | 5583549 (34.90%) | 8596523 (53.73%) | 1819928 (11.37%) | 65.10% |
| IL1_CCGTCC_L006_R1_003 | 841266 (34.98%) | 1293835 (53.79%) | 270030 (11.23%) | 65.02% |
| IL2_GTCCGC_L006_R1_001 | 6193180 (38.71%) | 8675585 (54.22%) | 1131235 (7.07%) | 61.29% |
| IL2_GTCCGC_L006_R1_002 | 6276215 (39.23%) | 8611190 (53.82%) | 1112595 (6.95%) | 60.77% |
| IL2_GTCCGC_L006_R1_003 | 960406 (39.28%) | 1317578 (53.89%) | 166739 (6.82%) | 60.72% |
| IL3_GTGAAA_L006_R1_001 | 5492271 (34.33%) | 8675616 (54.22%) | 1832113 (11.45%) | 65.67% |
| IL3_GTGAAA_L006_R1_002 | 5591387 (34.95%) | 8610799 (53.82%) | 1797814 (11.24%) | 65.05% |
| IL3_GTGAAA_L006_R1_003 | 1529046 (34.59%) | 2394873 (54.17%) | 496805 (11.24%) | 65.41% |
| IP1_ATCACG_L007_R1_001 | 6630907 (41.44%) | 8873936 (55.46%) | 495157 (3.09%) | 58.56% |
| IP1_ATCACG_L007_R1_002 | 6674407 (41.72%) | 8834352 (55.21%) | 491241 (3.07%) | 58.28% |
| IP1_ATCACG_L007_R1_003 | 298541 (43.81%) | 363473 (53.34%) | 19429 (2.85%) | 56.19% |
| IP2_TTAGGC_L007_R1_001 | 6779722 (42.37%) | 8720577 (54.50%) | 499701 (3.12%) | 57.63% |
| IP2_TTAGGC_L007_R1_002 | 6829776 (42.69%) | 8677273 (54.23%) | 492951 (3.08%) | 57.31% |
| IP2_TTAGGC_L007_R1_003 | 2185972 (42.92%) | 2750689 (54.01%) | 156291 (3.07%) | 57.08% |
| IP3_ACTTGA_L007_R1_001 | 6640446 (41.50%) | 8820726 (55.13%) | 538828 (3.37%) | 58.50% |
| IP3_ACTTGA_L007_R1_002 | 6689561 (41.81%) | 8777338 (54.86%) | 533101 (3.33%) | 58.19% |
| IP3_ACTTGA_L007_R1_003 | 1856074 (42.14%) | 2403837 (54.58%) | 144230 (3.27%) | 57.86% |

**Table S20. STAR mapping statistics for RNA-Seq data.** Reads were either uniquely mapped, multiply mapped, or unmapped.

| Category | Uniquely mapped | Mapped to multiple loci | Mapped to too many loci | Unmapped: too short | Unmapped: other |
| --- | --- | --- | --- | --- | --- |
| HA1_AGTCAA_L008_R1_001 | 14595930 (91.22%) | 876646 (5.47%) | 11105 (0.06%) | 509925 (3.18%) | 6394 (0.03%) |
| HA1_AGTCAA_L008_R1_002 | 14576753 (91.1%) | 875017 (5.46%) | 10953 (0.06%) | 532466 (3.32%) | 4811 (0.03%) |
| HA1_AGTCAA_L008_R1_003 | 3954575 (91.15%) | 235478 (5.42%) | 2907 (0.06%) | 144027 (3.31%) | 1301 (0.02%) |
| HA2_AGTTCC_L008_R1_001 | 14624232 (91.4%) | 855486 (5.34%) | 8391 (0.05%) | 507092 (3.16%) | 4799 (0.02%) |
| HA2_AGTTCC_L008_R1_002 | 14601095 (91.25%) | 855814 (5.34%) | 8375 (0.05%) | 529913 (3.31%) | 4803 (0.03%) |
| HA2_AGTTCC_L008_R1_003 | 2898331 (91.32%) | 166398 (5.24%) | 1615 (0.05%) | 106350 (3.35%) | 952 (0.02%) |
| HA3_ATGTCA_L008_R1_001 | 14542777 (90.89%) | 939581 (5.87%) | 9457 (0.05%) | 500195 (3.12%) | 7990 (0.04%) |
| HA3_ATGTCA_L008_R1_002 | 10658687 (90.72%) | 686227 (5.84%) | 6874 (0.05%) | 390648 (3.32%) | 5866 (0.04%) |
| HL1_CGATGT_L006_R1_001 | 14357459 (89.73%) | 1204041 (7.52%) | 21626 (0.13%) | 412082 (2.57%) | 4792 (0.02%) |
| HL1_CGATGT_L006_R1_002 | 13987114 (89.5%) | 1188833 (7.6%) | 20804 (0.13%) | 426118 (2.72%) | 4683 (0.02%) |
| HL2_TGACCA_L006_R1_001 | 14589259 (91.18%) | 951642 (5.94%) | 17914 (0.11%) | 436390 (2.72%) | 4795 (0.02%) |
| HL2_TGACCA_L006_R1_002 | 14540939 (90.88%) | 970597 (6.06%) | 17599 (0.1%) | 466060 (2.91%) | 4805 (0.03%) |
| HL2_TGACCA_L006_R1_003 | 799343 (90.9%) | 51274 (5.83%) | 951 (0.1%) | 27454 (3.12%) | 263 (0.02%) |
| HL3_ACAGTG_L006_R1_001 | 14521795 (90.76%) | 993853 (6.21%) | 20913 (0.13%) | 458645 (2.86%) | 4794 (0.02%) |
| HL3_ACAGTG_L006_R1_002 | 14482613 (90.51%) | 1014167 (6.33%) | 20112 (0.12%) | 478309 (2.98%) | 4799 (0.02%) |
| HL3_ACAGTG_L006_R1_003 | 4526864 (90.85%) | 307183 (6.16%) | 6477 (0.12%) | 140453 (2.81%) | 1494 (0.02%) |
| HP1_GCCAAT_L007_R1_001 | 15013468 (93.83%) | 538841 (3.36%) | 14844 (0.09%) | 429641 (2.68%) | 3206 (0.02%) |
| HP1_GCCAAT_L007_R1_002 | 15002642 (93.76%) | 536451 (3.35%) | 14703 (0.09%) | 443005 (2.76%) | 3199 (0.01%) |
| HP1_GCCAAT_L007_R1_003 | 6905319 (93.62%) | 249599 (3.38%) | 6658 (0.09%) | 212405 (2.87%) | 1475 (0.01%) |
| HP2_CAGATC_L007_R1_001 | 14984209 (93.65%) | 564293 (3.52%) | 14277 (0.08%) | 434018 (2.71%) | 3203 (0.02%) |
| HP2_CAGATC_L007_R1_002 | 14962594 (93.51%) | 564822 (3.53%) | 14548 (0.09%) | 454833 (2.84%) | 3203 (0.02%) |
| HP2_CAGATC_L007_R1_003 | 1353457 (93.06%) | 51356 (3.53%) | 1297 (0.08%) | 47941 (3.29%) | 291 (0.02%) |
| HP3_CTTGTA_L007_R1_001 | 14884512 (93.02%) | 605462 (3.78%) | 15271 (0.09%) | 491553 (3.07%) | 3202 (0.02%) |
| HP3_CTTGTA_L007_R1_002 | 14865009 (92.9%) | 605892 (3.78%) | 15079 (0.09%) | 510817 (3.19%) | 3203 (0.02%) |
| HP3_CTTGTA_L007_R1_003 | 1668396 (92.63%) | 67473 (3.74%) | 1580 (0.08%) | 63283 (3.51%) | 361 (0.02%) |
| IA1_GATCAG_L008_R1_001 | 14708426 (91.92%) | 770401 (4.81%) | 19616 (0.12%) | 496750 (3.1%) | 4807 (0.03%) |
| IA1_GATCAG_L008_R1_002 | 11811824 (91.78%) | 618294 (4.8%) | 15637 (0.12%) | 419513 (3.25%) | 3861 (0.03%) |
| IA2_TAGCTT_L008_R1_001 | 14471719 (90.44%) | 831834 (5.19%) | 22028 (0.13%) | 668026 (4.17%) | 6393 (0.03%) |
| IA2_TAGCTT_L008_R1_002 | 14450469 (90.31%) | 830542 (5.19%) | 22031 (0.13%) | 690564 (4.31%) | 6394 (0.03%) |
| IA2_TAGCTT_L008_R1_003 | 2546897 (90.27%) | 144594 (5.12%) | 3770 (0.13%) | 125026 (4.43%) | 1129 (0.04%) |
| IA3_GGCTAC_L008_R1_001 | 14085673 (88.03%) | 911597 (5.69%) | 17455 (0.1%) | 977278 (6.1%) | 7997 (0.04%) |
| IA3_GGCTAC_L008_R1_002 | 14071319 (87.94%) | 910565 (5.69%) | 17254 (0.1%) | 992868 (6.2%) | 7994 (0.04%) |
| IA3_GGCTAC_L008_R1_003 | 5785818 (87.9%) | 374266 (5.68%) | 7107 (0.1%) | 411938 (6.25%) | 2632 (0.03%) |
| IL1_CCGTCC_L006_R1_001 | 13897967 (86.86%) | 1641648 (10.26%) | 40679 (0.25%) | 413298 (2.58%) | 6408 (0.04%) |
| IL1_CCGTCC_L006_R1_002 | 13858927 (86.61%) | 1656611 (10.35%) | 39604 (0.24%) | 436857 (2.73%) | 8001 (0.05%) |
| IL1_CCGTCC_L006_R1_003 | 2091770 (86.97%) | 242260 (10.07%) | 5739 (0.23%) | 64397 (2.67%) | 965 (0.04%) |
| IL2_GTCCGC_L006_R1_001 | 14487730 (90.54%) | 1101502 (6.88%) | 26937 (0.16%) | 377434 (2.35%) | 6397 (0.03%) |
| IL2_GTCCGC_L006_R1_002 | 14440385 (90.25%) | 1124187 (7.02%) | 25795 (0.16%) | 403232 (2.52%) | 6401 (0.04%) |
| IL2_GTCCGC_L006_R1_003 | 2216157 (90.65%) | 164639 (6.73%) | 4009 (0.16%) | 58944 (2.41%) | 974 (0.03%) |
| IL3_GTGAAA_L006_R1_001 | 13967949 (87.29%) | 1614347 (10.08%) | 34546 (0.21%) | 376745 (2.35%) | 6413 (0.04%) |
| IL3_GTGAAA_L006_R1_002 | 13931259 (87.07%) | 1624531 (10.15%) | 33591 (0.2%) | 402630 (2.51%) | 7989 (0.04%) |
| IL3_GTGAAA_L006_R1_003 | 3866406 (87.46%) | 439203 (9.93%) | 9554 (0.21%) | 103794 (2.34%) | 1767 (0.03%) |
| IP1_ATCACG_L007_R1_001 | 14913907 (93.21%) | 571951 (3.57%) | 15282 (0.09%) | 494063 (3.08%) | 4797 (0.02%) |
| IP1_ATCACG_L007_R1_002 | 14894118 (93.08%) | 570904 (3.56%) | 15083 (0.09%) | 515096 (3.21%) | 4799 (0.02%) |
| IP1_ATCACG_L007_R1_003 | 627935 (92.14%) | 23942 (3.51%) | 576 (0.08%) | 28786 (4.22%) | 204 (0.02%) |
| IP2_TTAGGC_L007_R1_001 | 14601104 (91.25%) | 566110 (3.53%) | 17491 (0.1%) | 810499 (5.06%) | 4796 (0.02%) |
| IP2_TTAGGC_L007_R1_002 | 14582057 (91.13%) | 562874 (3.51%) | 17609 (0.11%) | 832665 (5.2%) | 4795 (0.02%) |
| IP2_TTAGGC_L007_R1_003 | 4633979 (90.98%) | 180845 (3.55%) | 5691 (0.11%) | 270909 (5.31%) | 1528 (0.03%) |
| IP3_ACTTGA_L007_R1_001 | 14841936 (92.76%) | 603422 (3.77%) | 13483 (0.08%) | 537957 (3.36%) | 3202 (0.02%) |
| IP3_ACTTGA_L007_R1_002 | 14824033 (92.65%) | 601016 (3.75%) | 13446 (0.08%) | 558306 (3.48%) | 3199 (0.01%) |
| IP3_ACTTGA_L007_R1_003 | 4075163 (92.53%) | 165259 (3.75%) | 3638 (0.08%) | 159199 (3.61%) | 882 (0.02%) |
